# Soybean AROGENATE DEHYDRATASES (GmADTs): involvement in the cytosolic isoflavonoid metabolon or trans-organelle continuity?

**DOI:** 10.3389/fpls.2024.1307489

**Published:** 2024-01-23

**Authors:** Emily J. Clayton, Nishat S. Islam, Kelsey Pannunzio, Kuflom Kuflu, Ramtin Sirjani, Susanne E. Kohalmi, Sangeeta Dhaubhadel

**Affiliations:** ^1^ London Research and Development Centre, Agriculture and Agri-Food Canada, London, ON, Canada; ^2^ Department of Biology, University of Western Ontario, London, ON, Canada

**Keywords:** soybean, arogenate dehydratase, isoflavone synthase, phenylalanine, isoflavonoid, metabolon, specialized metabolites

## Abstract

Soybean (*Glycine max*) produces a class of phenylalanine (Phe) derived specialized metabolites, isoflavonoids. Isoflavonoids are unique to legumes and are involved in defense responses *in planta*, and they are also necessary for nodule formation with nitrogen-fixing bacteria. Since Phe is a precursor of isoflavonoids, it stands to reason that the synthesis of Phe is coordinated with isoflavonoid production. Two putative AROGENATE DEHYDRATASE (ADT) isoforms were previously co-purified with the soybean isoflavonoid metabolon anchor ISOFLAVONE SYNTHASE2 (GmIFS2), however the *GmADT* family had not been characterized. Here, we present the identification of the nine member *GmADT* family. We determined that the GmADTs share sequences required for enzymatic activity and allosteric regulation with other characterized plant ADTs. Furthermore, the GmADTs are differentially expressed, and multiple members have dual substrate specificity, also acting as PREPHENATE DEHYDRATASES. All GmADT isoforms were detected in the stromules of chloroplasts, and they all interact with GmIFS2 in the cytosol. In addition, GmADT12A interacts with multiple other isoflavonoid metabolon members. These data substantiate the involvement of GmADT isoforms in the isoflavonoid metabolon.

## Introduction

Soybean (*Glycine max* [L.] Merr) is an important grain legume grown worldwide. Soybean seeds are high in oil and protein content, making them a nutrient-rich food source for both livestock and humans (Hill and Breidenbach, 1974; Messina, 2010). As one of the most cultivated oilseed crops, agricultural waste from soybean harvest has potential applications as a biofuel source (Yong and Wu, 2022). Soybeans also produce a legume unique class of specialized metabolites called isoflavonoids. Isoflavonoids are important signaling molecules, as they are essential for interaction between legumes and nitrogen-fixing bacteria (Phillips and Kapulnik, 1995; Subramanian et al., 2006; Hassan and Mathesius, 2012). Isoflavonoid glyceollins act as phytoalexins and provide defense against abiotic and biotic stress, such as resistance against the soil-borne pathogen *Phytophthora sojae* (Subramanian et al., 2006; Lozovaya et al., 2007), that cause massive crop losses (Bradley et al., 2021; Chandra et al., 2022). Furthermore, there is some evidence suggesting that isoflavonoids such as genistein and glyceollin have health benefits (Lamartiniere, 2000; Dixon and Ferreria, 2002; Sarkar and Li, 2003; Cederroth and Nef, 2009; Messina, 2010; Bhat et al., 2021). As such, isoflavonoids have been the target of traditional breeding and metabolic engineering to improve cultivar resistance to biotic and abiotic stress (Scott et al., 2021; Yousefi-Taemeh et al., 2021).

**Figure 1:**
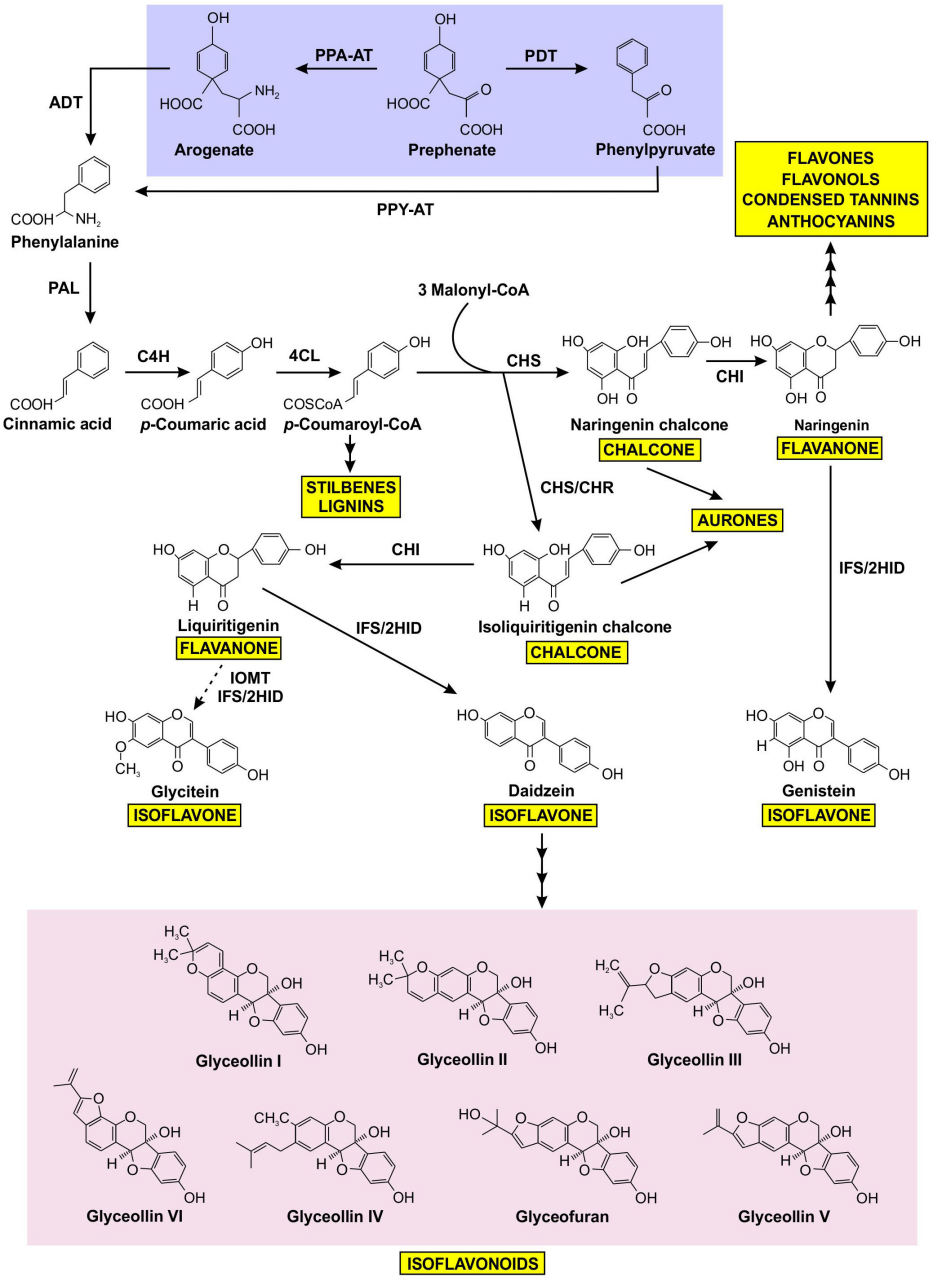
Prephenate from the shikimate pathway uses two different mechanisms to produce phenylalanine, which is subsequently channeled into the production of a variety of phenylpropanoids, including isoflavonoids. Phenylalanine produced by the arogenate and phenylpyruvate branches are highlighted in purple, isoflavonoids are highlighted in pink, and other phenylpropanoid end products are highlighted in yellow. The dotted arrow represents speculative steps, and multiple arrows indicate two or more steps in the pathway. PPA-AT, prephenate-aminotransferase; PDT, prephenate dehydratase; PPY-AT, phenylpyruvate aminotransferase; PPA-AT, prephenate aminotransferase; ADT, arogenate dehydratase; PAL, phenylalanine ammonia lyase; C4H, cinnamate 4-hydroxylase; 4CL, 4–coumarate: CoA ligase; CHS, chalcone synthase; CHR, chalcone reductase; CHI, chalcone isomerase; IFS, isoflavone synthase; 2HID, 2-hydroxyisoflavanone dehydratase; I2’H, isoflavone 2’-hydroxylase; IOMT, isoflavone O–methyltransferase.

**Figure 2:**
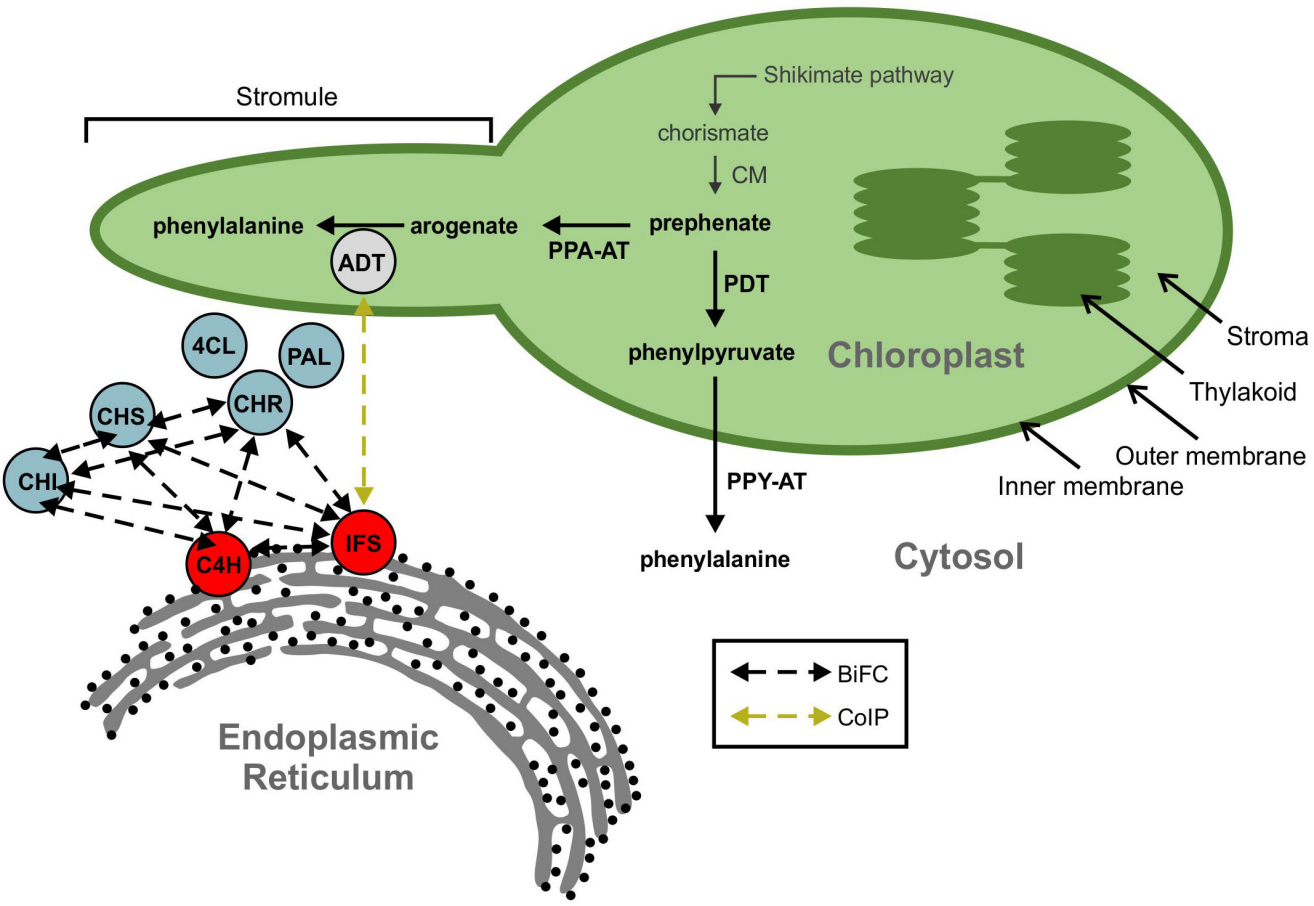
A model showing ADT interaction with the isoflavonoid metabolon. The isoflavonoid metabolon is made up of cytoplasm-localized enzymes (blue) anchored to the cytosolic face of the ER membrane through interaction with cytochrome P450 enzymes, IFS and C4H (red). Protein-protein interactions between multiple members of the isoflavonoid metabolon have been determined by BiFC (black dashed arrows). An interaction between IFS and an ADT (grey) was also detected *via* Co-IP (yellow dashed arrow). ADT enzymes are involved in phenylalanine biosynthesis and are localized to the chloroplast along with the shikimate pathway and other aromatic amino acid biosynthetic machinery. Subcellular compartments are indicated by grey fonts. ADT, arogenate dehydratase; PAL, phenylalanine ammonia lyase; 4CL, 4-coumarate-CoA ligase; C4H, cinnamate 4-hydroxylase; CHI, chalcone isomerase; CHR, chalcone reductase; CHS, chalcone synthase; CM, chorismite mutase; IFS, isoflavone synthase; PDT, prephenate dehydratase; PPA-AT, prephenate aminotransferase; PPY-A, phenylpyruvate aminotransferase.

The biosynthesis of isoflavonoids is a complex process that is derived from the phenylpropanoid metabolism (Winkel, 2001), downstream from the synthesis of the aromatic amino acid phenylalanine (Phe). As shown in **Figure 1**, the first committed step of (iso)flavonoid biosynthesis is the action of CHALCONE SYNTHASE (CHS) to synthesize a chalcone scaffold from which all (iso)flavonoids are built (Winkel, 2006; Dastmalchi and Dhaubhadel, 2014). The key branch point of isoflavonoid synthesis from the flavonoid biosynthetic pathway is catalyzed by the cytochrome P450 enzyme ISOFLAVONE SYNTHASE (IFS) where naringenin and liquiritigenin are converted to isoflavones genistein and daidzein, respectively (Winkel, 2006; Dastmalchi and Dhaubhadel, 2014; García-Calderón et al., 2020). Many of the enzymes involved in (iso)flavonoid biosynthesis have been shown to form protein-protein interactions (Winkel, 2004; Jorgensen et al., 2005; Dastmalchi et al., 2016). The phenylpropanoid pathway was first proposed to form a multienzyme metabolon by Helen Stafford (1974) and further confirmed by Hrazdina and Wagner (1985), as an explanation for the efficiency of substrate channeling in specialized metabolite biosynthesis. Further evidence demonstrated that the metabolon includes enzymes involved in flavonoid synthesis, such as CHS, CHALCONE ISOMERASE (CHI), and FLAVONOL SYNTHASE (FLS) (Winkel, 2004; Nakayama et al., 2019). In soybean, this metabolon also includes key isoflavonoid biosynthetic enzymes CHALCONE REDUCTASE (CHR) and IFS (**Figure 2**) (Dastmalchi et al., 2016). The isoflavonoid biosynthetic metabolon is associated with the cytosolic surface of the endoplasmic reticulum (ER), anchored by the two ER membrane cytochrome P450 monooxygenase enzymes CINNAMATE 4-HYDROXYLASE (C4H) and IFS (Jorgensen et al., 2005; Dastmalchi et al., 2016). To date, the flavonoid metabolon has been identified in a wide range of plant species (Owens et al., 2008; Crosby et al., 2011; Waki et al., 2016; Nakayama et al., 2019), conferring a variety of advantages such as metabolic channeling of substrates, or the sequestering of toxic intermediates (Moller, 2010; Pareek et al., 2021; Zhang and Fernie, 2021).

When characterizing the soybean isoflavonoid metabolon, we detected an interaction between GmIFS2 and two putative AROGENATE DEHYDRATASES (ADTs), Glyma.12G181800.1 and Glyma.13G319000.1 (Dastmalchi et al., 2016). ADTs catalyze the final step in Phe biosynthesis via the arogenate pathway (Maeda and Dudareva, 2012) in which prephenate is converted to arogenate by a PREPHENATE AMINOTRANSFERASE (PPA-AT; (Graindorge et al., 2010; Maeda et al., 2011), followed by a decarboxylation/dehydration to Phe, catalyzed by an ADT (Jung et al., 1986; Ehlting et al., 2005; Cho et al., 2007). The interaction between ADT and IFS observed in our previous work was an unexpected finding (Dastmalchi et al., 2016), as ADTs have been shown to localize within the chloroplasts in other plant species, consistent with the chloroplastic localization of shikimate and arogenate pathway enzymes (Cho et al., 2007; Rippert et al., 2009; Maeda et al., 2010; El-Azaz et al., 2016). However, a second Phe biosynthetic route, the prephenate pathway, had been described where prephenate is first decarboxylated/dehydrated into phenylpyruvate by a PREPHENATE DEHYDRATASE (PDT; (Cotton and Gibson, 1965), which is then transaminated into Phe by the action of a PHENYLPYRUVATE AMINOTRANSFERASE (PPY-AT; (Cotton and Gibson, 1965; Fazel et al., 1980). While prephenate pathway is predominantly used by microbes, there was evidence for existence of the prephenate pathway in plants. PPY-ATs had been identified in several plant species (Watanabe et al., 2002; Kaminaga et al., 2006; Warpeha et al., 2006), and PDT activity had been reported in *Petunia hybrida*, *Arabidopsis*, *Oryza sativa* and *Pinus pinaster*, though the dual ADT/PDT enzymes have a preference for arogenate over prephenate (Cho et al., 2007; Yamada et al., 2008; Maeda et al., 2010; El-Azaz et al., 2016; El-Azaz et al., 2022). Recently, cytosolic Phe biosynthesis *via* the prephenate pathway was reported in *Petunia x hybrida* (Yoo et al., 2013; Qian et al., 2019) and is expected to act in parallel with the arogenate pathway. As the isoflavonoid metabolon is anchored to the cytosolic face of the ER, the two GmADTs identified as part of the isoflavonoid metabolon (Dastmalchi et al., 2016) could be part the cytosolic Phe synthesis route, and may have PDT activity. As most plant genomes encode at least two, if not more *ADT* genes, there are likely many more than two GmADT isoforms present in soybean as it is a paleopolyploid. These isoforms may also interact with the isoflavonoid metabolon. However, the complete GmADT family has not been characterized.

Here we identified additional ADT family members in soybean and describe their gene structure, phylogeny, tissue-specific gene expression and subcellular localization. We demonstrate that some members of the GmADT family contain PDT activity in yeast complementation analysis. Despite the fact that all GmADTs were detected in the chloroplast, we confirmed their ability to interact with GmIFS2 and multiple other isoflavonoid metabolon enzymes *in planta*. Together, these data suggest that members of the GmADT family are associating with the isoflavonoid metabolon, and those with PDT activity could be supplying Phe to the metabolon through a cytosolic prephenate pathway.

## Materials and methods

### Plant materials and growth conditions

*Nicotiana benthamiana* was grown on PRO-MIX^®^ BX MYCORRHIZAETM soil (Rivière-du-Loup, Canada) in a growth room set to 16 h light at 24°C and 8 h dark at 20°C with 60% relative humidity and a light intensity of 80-100 μmol m^-2^s^-1^.

Soybean cultivar Williams 82 seeds were planted in sterile pots containing PRO-MIX^®^ BX MYCORRHIZAE™ soil (Premier Tech Home and Garden, Rivière-du-Loup, QC) and maintained in a growth room under a 16 h light and 8 h dark cycle at 24°C with 60-70% humidity and a light intensity of 250 μmol m^-2^s^-1^. The plants were watered with a fertilizer solution containing nitrogen-phosphorus-potassium (20-8-20). At the flowering stage of soybean plants, stem, leaf, root, and flower tissue were harvested, frozen in liquid nitrogen, and stored at -80°C.

### *In silico* and phylogenetic analyses

Candidate soybean *ADTs* (*GmADT*s) were identified by mining the soybean genome in Phytozome 13 (https://phytozome-next.jgi.doe.gov/). Two previously identified soybean ADTs Glyma.13G319000.1 and Glyma.12G181800.1 (GmADT13A and GmADT12A, **Table 1**) (Dastmalchi et al., 2016) were used in BLAST searches against the soybean genome database (Glycine max Wm82.a4.v1). Each unique gene identified from the two initial input sequences was used in BLAST search again to look for all possible GmADTs. Multiple sequence alignments were performed using protein sequences of ADTs in Clustal Omega (Sievers et al., 2011), and visualized in boxshade using pyBoxshade (https://github.com/mdbaron42/pyBoxshade). TargetP was used for subcellular localization and cleavage site prediction (Emanuelsson et al., 2007). The gene and transcript data (in gff3 format) for *GmADT*s were retrieved from Phytozome 13 for Wm82.a4.v1 genome assembly and the gene structure model was generated using TBtools (Chen et al., 2020).

**Table 1:** Characteristics of putative *ADT* gene family members in soybean.

Gene name	Locus name	Locus range	Coding sequence length (bp)	Predicted protein size (kDa)	Splice variants	Predicted subcellular localization
*GmADT9*	Glyma.09G004200	Gm09:331514.337738	1215	44.5	2	Other
*GmADT11A*	Glyma.11G189100	Gm11:16210640.16212382	1287	46.9	1	Chloroplast
*GmADT11B*	Glyma.11G151288	Gm11:11415143.11420316	1158	42.7	1	Other
*GmADT12A*	Glyma.12G181800	Gm12:35716180.35718032	1278	46.2	1	Chloroplast
*GmADT12B*	Glyma.12G085500	Gm12:6875420.6877191	1287	46.8	1	Chloroplast
*GmADT12C*	Glyma.12G193000	Gm12:36904068.36910226	1155	42.8	1	Chloroplast
*GmADT12D*	Glyma.12G072500	Gm12:5330594.5336280	933	33.8	1	Chloroplast
*GmADT13A*	Glyma.13G319000	Gm13:40728761.40730567	1275	46.1	1	Chloroplast
*GmADT13B*	Glyma.13G309300	Gm13:39881239.39884055	645	23.1	1	Other
*GmADT17*	Glyma.17G012600	Gm17:970102.977629	1200	43.7	1	Chloroplast

For phylogenetic analysis, the predicted transit peptide sequences were removed from plant ADTs and putative GmADTs according to Cho et al. (2007). The chorismate mutase domain of the *E. coli* P-protein was removed according to Zhang et al. (1998). The mature protein sequences were aligned using ClustalW and the tree was constructed with the bootstrap set to 1,000 replicates using MEGAX software (Kumar et al., 2018).

### Gene expression analysis and heat map generation

Soybean RNA-seq data was retrieved from Phytozome 13 database with expression values in FPKM (Wang et al., 2019). A heatmap was generated using log_2_-transformed normalized transcript abundance values using TBtools (Chen et al., 2020). Gene cluster in the Newick tree was generated in MEGAX and imported into the heatmap.

### RNA extraction and reverse transcription-PCR

Total RNA was extracted from soybean tissues (50-70 mg) using the RNeasy plant Mini kit (Qiagen). An on column DNase I (Promega) treatment was performed prior to RNA elution from each sample. Total RNA (1 μg) was used to synthesize cDNA using oligo dT primers and SuperScript IV First Strand Synthesis System (Thermofisher) as per manufacturer’s instructions.

### Cloning of *GmADT*s

The coding regions of *GmADTs* were amplified using RT-PCR with gene-specific primers (**Supplementary Table 1**) and cloned into pDONR-Zeo (Invitrogen) using BP clonase^®^ (Invitrogen), followed by transformation into *E. coli* DH5α via electroporation. The recombinant entry clones were confirmed by sequencing and then recombined with the destination vectors pEarleyGate101 (pEG101) for subcellular localization (Earley et al., 2006) and pEarleyGate201-YN and pEarleyGate202-YC for *in planta* protein-protein interaction assays (Lu et al., 2010) in an LR recombination reaction (Invitrogen). The expression clones were transformed into *Agrobacterium tumefaciens* GV3101. GmIFS2 (Glyma.13G173500) in pEarleyGate201-YN and pEarleyGate202-YC were obtained from Dastmalchi et al. (2016).

For the PDT assay, each of the *GmADT*s with a 6×His-C-terminal fusion was recombined into the destination vector pAG423GAL-ccdB-ECFP (Addgene plasmid # 14173; http://n2t.net/addgene:14173; RRID : Addgene_14173) using Gateway technology as described above and transformed into *Saccharomyces cerevisiae pha2* (Ackerman et al., 1992) using Frozen-EZ Yeast Transformation II™ kit (Zymo Research). Transformants were screened on minimal synthetic dextrose (SD)/-His plates.

### Confocal microscopy

For subcellular localization, *A. tumefaciens* harboring pEG101 containing *GmADT*s were transformed into *N. benthamiana* leaves by infiltration as described by Sparkes et al. (2006). For protein-protein interaction by BiFC, fusions containing YN and YC fragments of YFP were co-infiltrated into *N. benthamiana* leaves in a 1:1 (v/v) mixture as described before. The protein expression was visualized 48 h post-infiltration by using the Olympus FV1000 confocal microscope under a 60× water immersion objective lens. For YFP visualization, the excitation wavelength was set to 514 nm and emission was collected at 520-550 nm. For chloroplast-visualization, the natural auto-fluorescence produced by chlorophyll was harnessed by exciting the chlorophyll at 600 nm and emission was collected at 640-700 nm.

### *pha2* complementation assay

For the *pha2* complementation assay (Bross et al., 2011), yeast cultures were grown in appropriately supplemented liquid raffinose media overnight at 30°C with shaking. Each culture diluted in double distilled water to a final density of 5 x 10^4^ cells/mL, and 10 μL of cells were spotted on appropriate selection media. SD plates prepared with different carbon sources (glucose, galactose, and raffinose) were used. The *GAL1* promoter in the destination vectors is expressed or repressed by galactose or glucose, respectively, whereas raffinose has no influence on the regulation of the promoter (St John and Davis, 1981; Lohr et al., 1995). Plates of each carbon source were made either with a -histidine (-His), or a -histidine-phenylalanine (-His-Phe) dropout powder. The lack of His selects for the presence of the ADT expression vector, while the lack of Phe selects for PDT activity when ADT proteins are induced. Negative (untransformed *pha2* and empty destination vector) and positive (WT AtADT2) controls were spotted on every plate, and all complementation tests were repeated at least 3 times. Images were taken with a digital camera (Canon EOS 70D) six and thirteen days after spotting, and a single representative image of each construct is shown.

### Western blotting

Yeast cultures were grown overnight in glucose media, washed twice with double distilled water, then grown overnight to an OD_600_ of 0.6-0.8 in glucose media as a negative control, or in galactose media to induce ADT expression. Total protein was extracted using the yeast alkaline lysis method (Kushnirov, 2000). Protein extracts were then size separated on an SDS-PAGE gel (6% stacking gel, 12% separating gel). Following electrophoresis, the resolved proteins were visualized with Coomassie Brilliant Blue. Gels were incubated at room temperature for 30 minutes with shaking, and then destained for 2-3 hrs to remove background stain before an image was taken.

GmADT-6×His and CHR14-6xHis fusion proteins were detected using Western blot analysis with a monoclonal mouse anti-His primary antibody (1:1200, Sigma, SAB1305538). A goat anti-mouse HRP conjugate secondary antibody (BioRad, 1706516) was used in all cases, and HRP activity was visualized using the Clarity ECL kit (BioRad, 1705061).

## Results

### The soybean genome contains 10 putative *GmADT* genes

To identify all the members of the *GmADT* gene family, we used *Glyma.12G181800* (*GmADT12A*) and *Glyma.13G319000* (*GmADT13A*) sequences as queries in a BLAST search in the *G. max* Wm82.a4.v1 genome database. These two searches identified 10 *GmADT*s. Each of these *GmADT*s were used separately as a query sequence in the BLAST search in the soybean genome database. This process was repeated until no new *ADT* was discovered (**Table 1**). The following nomenclature was developed: *GmADT*s were numbered according to the chromosome on which they are encoded and a letter (A, B, etc) was added if more than one *GmADT* was located on the same chromosome.

The 10 identified *ADT* loci in the soybean genome are distributed across five different chromosomes, with chromosome 12 containing four *GmADTs* (*GmADT12A, GmADT12B, GmADT12C* and *GmADT12D*) while chromosomes 11 (*GmADT11A, GmADT11B*) and 13 (*GmADT13A, GmADT13B*) each contain two *GmADTs*. Chromosomes 9 and 17 carry only one *GmADT* each (*GmADT9 and GmADT17*, **Table 1**). The putative *GmADT* genes encode proteins with a calculated molecular mass ranging from 23.1 to 46.9 kDa (**Table 1**).

**Figure 3:**
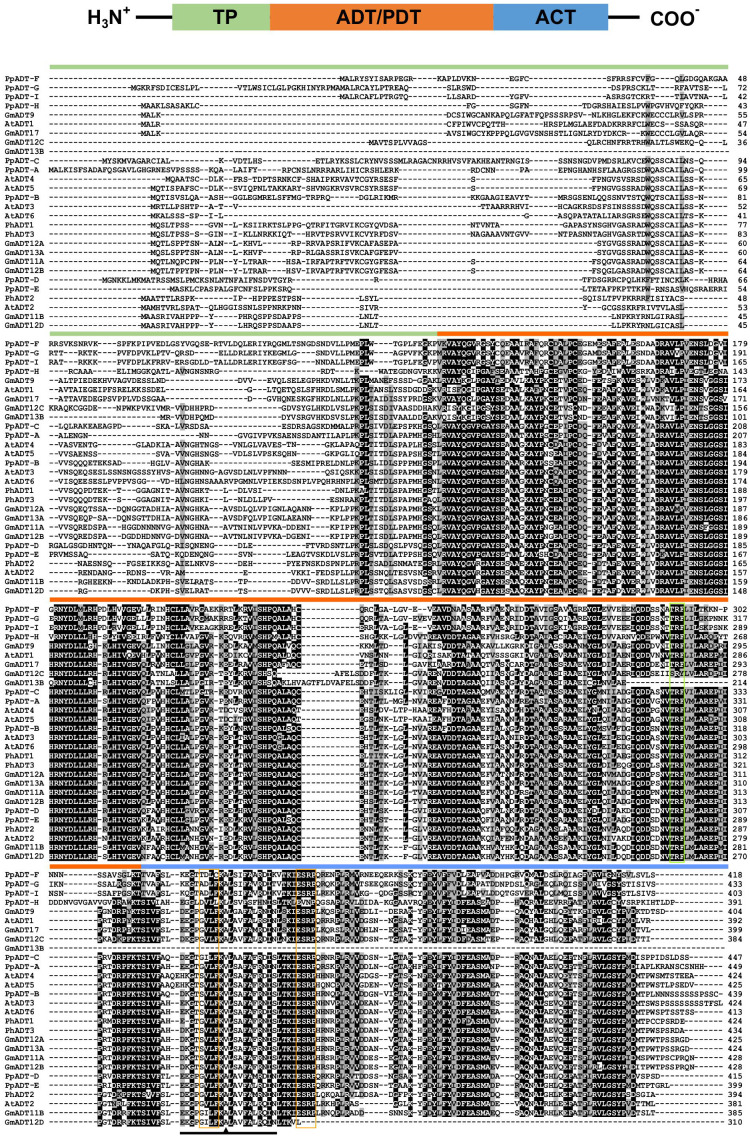
Protein domains and multiple sequence alignment of GmADTs and AtADTs. A schematic diagram (top) of predicted protein domains of ADTs include transit peptide (TP), catalytic ADT or PDT domain and regulatory ACT domain (modified from Cho et al., 2007). The amino acid sequences of the GmADTs, *Arabidopsis* ADTs (AtADT), *Petunia hybrida* (PhADTs) and *Pinus pinaster* (PpADTs) were aligned using ClustalO. Black shading indicates identical residues, grey shading indicates similar amino acid residues and dashed lines indicate gaps. Colored bars above the sequences indicate predicted protein domains color coded as shown on top. Green boxe indicate TRF motif. Conserved A (A314 in PpADT) residue conferring PDT activity is shown by the arrow head within the PAC domain (black line). Yellow boxes indicate GALV and ESRP domains.

A multiple sequence alignment of previously characterized ADTs from *Arabidopsis thaliana* (AtADTs), *Petunia x hybrida* (PhADTs) and *Pinus pinaster* (PpADTs) and the deduced amino acid sequences of GmADTs revealed that, similar to other plant ADTs, GmADTs also contain a putative N-terminal transit peptide, an internal catalytic domain, and a C-terminal ACT domain (**Figure 3**). As expected the transit peptide regions are highly variable while the catalytic and ACT domains of the GmADT, AtADT, PhADT and PpADT proteins exhibit substantial levels of sequence conservation. Comparing GmADT sequences, GmADT9 is the most varied member of the family and shares only 49.5-63.3% sequence identity with other isoforms at the amino acid level. A pairwise percentage identity of full-length GmADTs sequences at the amino acid and nucleotide levels varied from 49.5 to 96.2% and 50.6 to 94.8%, respectively (**Table 2**). Most GmADTs except GmADT12C and GmADT13B contain the conserved TRF triad (**Figure 3**, green box) in the catalytic domain that is critical for substrate binding and prephenate/arogenate catalysis (Zhang et al., 2000; Hsu et al., 2004; Tan et al., 2008). Instead of the TRF triad, GmADT12C contains an SRY sequence instead. In addition, key ligand binding motifs (ESRP and GALV) in the ACT domain (Pohnert et al., 1999; Tan et al., 2008) are also shared by the GmADTs and other ADTs. However, the C-terminal region of the catalytic domain and the entire ACT domain from GmADT13B, and the C-terminus of the ACT domain from GmADT12D are missing. As such, GmADT12D contains the GALV but lacks the ESRP regulatory motif due to C-terminal truncation. Soybean cultivar Williams 82 whole genome sequence has been reassembled multiple times, however, this discrepancy was observed consistently including in the most recent release *Glycine max* var. Williams 82-ISU-01 (https://phytozome-next.jgi.doe.gov/info/GmaxWm82ISU_01_v2_1). As GmADT13B lacks the entirety of the ACT domain, it is likely not allosterically regulated if it is a functional protein.

**Table 2:** Pairwise coding region and amino acid sequence comparisons of the soybean *GmADT* gene family.

Name	GmADT9	GmADT11A	GmADT11B	GmADT12A	GmADT12B	GmADT12C	GmADT12D	GmADT13A	GmADT13B	GmADT17
Amino acids										
*GmADT9*		49.49	52.85	49.75	49.75	54.35	53.4	50.13	54.73	65.33
*GmADT11A*	50.88		56.76	81.8	94.39	52.52	56.95	81.28	53.77	53.96
*GmADT11B*	57.2	53.3		56.38	55.97	56.82	95.48	56.38	51.52	55.71
*GmADT12A*	51.93	79.72	53.21		81.8	53.6	56.81	96.23	55	54.76
*GmADT12B*	50.63	93.94	52.77	79.8		53.05	57.62	81.04	53.77	54.22
*GmADT12C*	60.95	51.84	56.41	53.81	52.47		55.2	54.01	92.08	59.15
*GmADT12D*	57.17	51.75	93.89	51.75	51.43	53.86		57.14	52.79	56.12
*GmADT13A*	51.98	79.43	53.53	93.96	78.96	54.72	51.82		56	54.9
*GmADT13B*	61.22	51.14	55.76	53.33	51.8	94.75	55.95	53.82		57
*GmADT17*	76.72	53.46	58.67	53.37	53.55	63.85	57.85	52.91	63.71	
Nucleotides										

Originaly ADTs were thought to be monofunctional enzymes like bacterial PDTs. However it has been demonstrated that some ADT isozymes can also act as PDTs (Cho et al., 2007; Maeda et al., 2010; Bross et al., 2011; El-Azaz et al., 2016; Qian et al., 2019). ADT and PDT enzymes catalyze the same biochemical reaction (decarboxylation/dehydration) using very similar substrates, and many key catalytic and regulatory residues are conserved between them (Cho et al., 2007; Tan et al., 2008). Previous work to determine the sequences responsible for this dual substrate recognition in *Pinus pinaster* ADTs (PpADTs) identified a 22 amino acid region at the N-terminus of the ACT domain, termed the PDT activity conferring domain, or PAC domain (El-Azaz et al., 2016). Additionally, a critical alanine residue (Ala314 in PpADT-G) was to be sufficient to define prephenate substrate recognition in the PpADTs. Five GmADTs contain this alanine residue within the PAC domain: GmADT9, GmADT11B, GmADT12C, GmADT12D and GmADT17 (**Figure 3**), suggesting these isoforms are ADT/PDTs.

### *GmADT* gene structure and phylogenetic analysis

**Figure 4:**
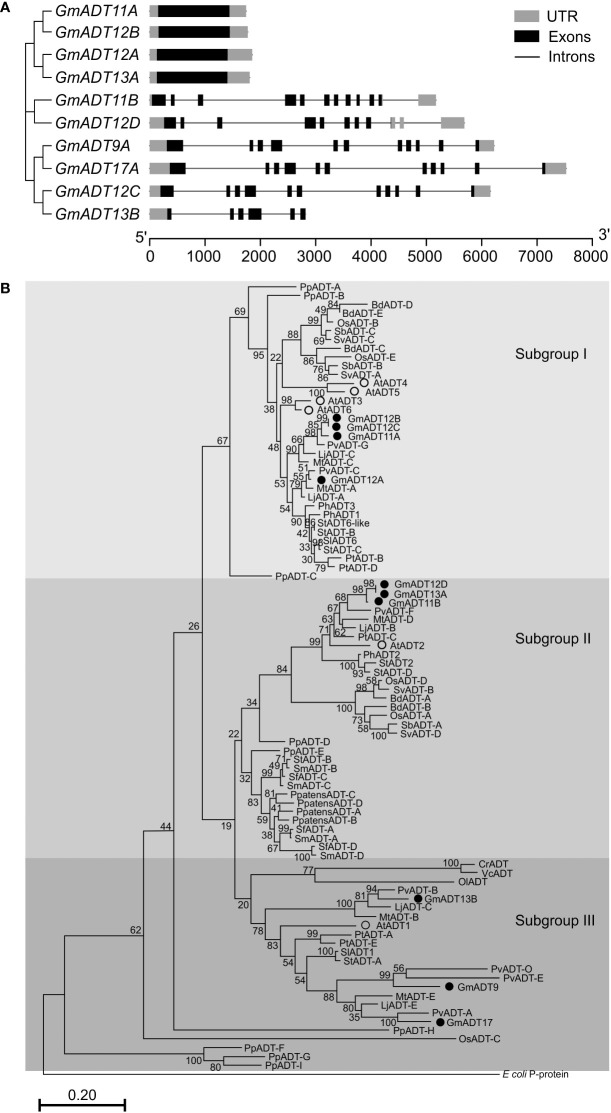
Gene structure and phylogenetic analysis of GmADTs. **(A)** Genomic structure of *GmADTs* were drawn to scale using gene models of *GmADT*s, retrieved from Phytozome 13 for soybean (*G. max* Wm82.a4.v1) genome and the gene cluster was imported from a neighbor-joining tree of *GmADT*s. **(B)** Mature protein sequences of GmADTs, AtADTs, PpADTs and PhADTs were identified using TargetP, aligned using ClustalΟ and a phylogenetic tree was constructed by neighbor-joining method using MEGAX. Bootstrap values (1000 replicates) are shown as percentages next to branch points. The PDT domain of the *E. coli* P-protein was included as an outgroup. At, *Arabidopsis thaliana;* Pp, *Pinus pinaster;* Ph, *Petunia hybrida.* Accession numbers: AtADT1, AT1G11790.1; AtADT2, AT3G07630.1; AtADT3, AT2G27820.1; AtADT4, AT3G44720.1; AtADT5, AT5G22630.1; AtADT6, AT1G08250.1; PhADT1, ACY79502.1; PhADT2, ACY79503.1; PhADT3, ACY79504.1; PpADTA, APA32582.1; PpADTB, APA32583.1; PpADTC, APA32584.1; PpADTD, APA32585.1; PpADTE, APA32586.1; PpADTF, APA32587.1; PpADTG, APA32588.1; PpADTH, APA32589.1; PpADTI, APA32590.1; *E. coli*, WP_115444483.1.

An analysis of *GmADT* gene structure indicated that four *GmADT*s (*GmADT11A*, *GmADT12A, GmADT12B* and *GmADT13A*) contained no introns in their open reading frame while six contained multiple introns (**Figure 4A**). *GmADT9*, *GmADT11B*, *GmADT12C* and *GmADT17* contained 10 introns of varying sizes ranging from 81 to 1444 nucleotides while *GmADT12D* and *GmADT13B* contained eight and five introns, respectively. Among all the *GmADT* family members, only *GmADT11B* and *GmADT12D* contained introns in its 3`UTR.

To illustrate the evolutionary relationship among soybean ADTs and other characterized ADT proteins, a phylogenetic analysis was performed using the predicted amino acid sequences of their mature proteins. As shown in **Figure 4B**, similar to AtADTs (Cho et al., 2007), soybean ADTs form three distinct subgroups. GmADT9, GmADT13B, and GmADT17 grouped in ‘subgroup III’ with AtADT1. Three GmADTs, GmADT11B, GmADT12D, and GmADT13A grouped into ‘subgroup II’ with AtADT2 and PhADT2 while four GmADTs, GmADT11A, GmADT12A, GmADT12B and GmADT12C, grouped in ‘Subgroup I’ with AtADT3, AtADT4, AtADT5 and AtADT6. Similar to other angiosperms, at least one GmADT isoform is present in each subgroup. In addition, GmADTs in each subgroup are clustered with ADT sequences with other legume species (*Medicago truncatula, Lotus japonicus*, and *Phaseolus vulgaris*). As observed for most of the gene families in soybean, GmADTs mostly group in pairs, consistent with its recent whole genome duplication (Schmutz et al., 2010; Yuan and Song, 2023). Subgroups II and III contain other plant ADTs that have been shown to act as PDTs (eg. AtADT1, AtADT2, PpADT-G, PhADT2).

### Expression analysis of *GmADT* genes

**Figure 5:**
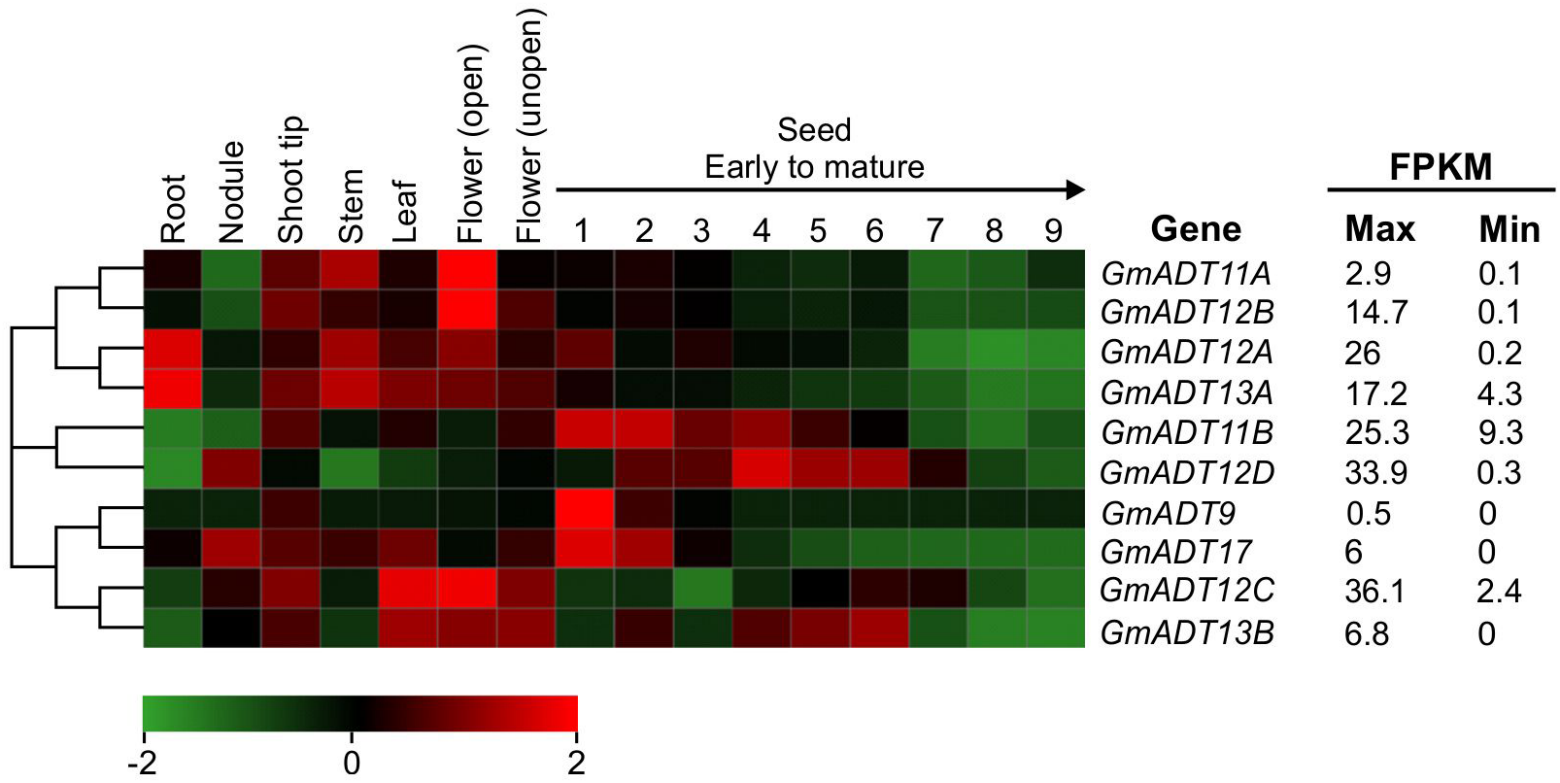
mRNA expression analysis of *GmADT*s. The transcriptome data of *GmADT* across different tissues were retrieved from Phytozome 13 database (Wang et al., 2019) for heatmap generation. The maximum (max) and minimum (min) expression values for each gene are shown and the gene cluster was imported from neighbor-joining tree generated with GmADT protein sequences in MEGAX. The black arrow on top of 1 to 9 indicates seed developmental stages from early (1) to mature (9). The color scale below the heatmap indicates transcript abundance values in log2 transformed across each row. Red and green indicating high and low levels of transcript abundance, respectively. FPKM, fragments per kilobase of transcript per million.

To determine the mRNA expression patterns of the *GmADT* gene family members in soybean tissues, we utilized the transcriptome dataset available in the public domain as a resource (Wang et al., 2019). As shown in **Figure 5**, the dataset obtained from Phytozome 13 database consisted of transcript accumulation in root, nodules, stem, leaf, flowers (open and unopen) and seed tissues collected during the development from early to mature seeds. The maximum fragments per kilobase of transcript per million mapped reads (FPKM) values of *GmADT*s varied from 0.5 (*GmADT9* in early seed development) to 36.1 (*GmADT12C* in flowers). While the majority of *GmADT*s were expressed in most of the tissue analyzed, each gene family member displayed a unique tissue-specific expression pattern. *GmADT12A* and *GmADT13A* transcripts accumulated to highest levels in root tissues while the transcripts of only *GmADT12D* and *GmADT17* were found in nodules. Transcript accumulation for *GmADT9, GmADT11B, GmADT12A, GmADT12D* and *GmADT17* was higher during early seed development whereas expression of *GmADT13B* was detected in leaf, flower and seed tissue at mid-maturation stage. Despite lacking the transit peptide and the ACT domain, the predicted *GmADT13B* transcript levels were detectable, thus potentially producing only a truncated protein (**Figure 3**; **Table 1**). Therefore, it is likely that GmADT13B is non-functional as ADT/PDT and was excluded from further characterization in this study.

### GmADTs primarily localize to chloroplasts

**Figure 6:**
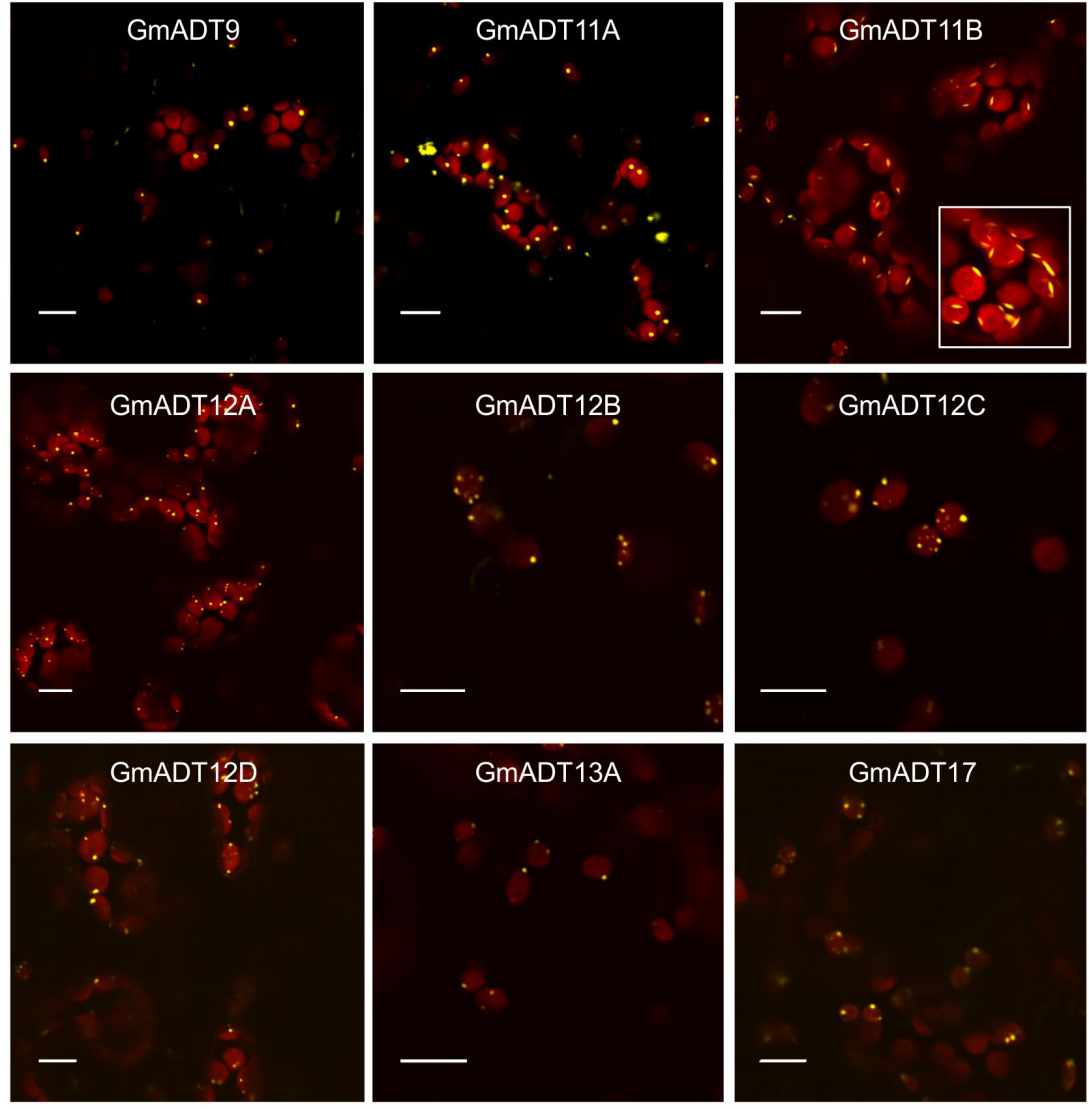
Subcellular localization of the GmADTs. A translational fusions of GmADT-YFP were transiently expressed in *N. benthamiana* leaf and visualized by confocal microscopy. Confirmation of localization was performed through co-localization of the GmADT-YFP fusion with the chloroplast autofluorescence (in red). Scale bars represent 10 µM.

Plant ADTs were previously reported to localize to the chloroplasts in multiple species including *Arabidopsis*, petunia and pine (Rippert et al., 2009; Maeda et al., 2010; El-Azaz et al., 2016; Bross et al., 2017). To confirm if the GmADTs also are chloroplast localized, we investigated the subcellular localization of the nine GmADTs that were initially annotated in Phytozome as full length ADTs (as predicted truncated protein, GmADT13B was excluded from further analysis). GmADTs were translationally fused to YFP, transiently expressed in *N. benthamiana* leaves, and visualized by confocal microscopy. The red auto-fluorescence generated by chlorophyll was used as a chloroplast marker. As shown in **Figure 6**, all nine GmADTs showed chloroplastic localization, specifically a stromule sub-plastidial localization. Stromules resemble tail-like or bead-like protrusions from the chloroplast body (Arimura et al., 2001; Pyke and Howells, 2002; Kwok and Hanson, 2004b; Hanson and Hines, 2018). This stromule sub-plastidial localization of the GmADTs is consistent with the reports for other plant ADTs (Maeda et al., 2010; El-Azaz et al., 2016; Bross et al., 2017). Furthermore, GmADT11B displayed additional thin, elongated sub-plastidial localization at the poles of the chloroplasts (**Figure 6**, inset), distinct from the other GmADTs but similar to those observed for AtADT2, an AtADT likely involved in chloroplast division (Abolhassani Rad, 2017).

### GmADTs interact with isoflavonoid metabolon enzymes

To determine if the GmADT and GmIFS2 interaction detected *via* Co-IP is actually occurring *in-planta*, we conducted Bimolecular Fluorescence Complementation (BiFC) assay to assess protein-protein interactions. In our BiFC system we used *N. benthamiana*, which synthesizes flavonoids but not isoflavonoids. However, we are introducing the key branch enzyme leading to isoflavonoid synthesis, GmISF2. The flavonoid synthesis machinery upstream of IFS2 is the same in both legumes and non legumes, allowing us to use the *N. benthamiana* transient expression system to assess GmADT interactions with GmIFS2. As we were expressing soybean genes in a heterologous system, all contructs were expessed using a 35S promoter to ensure comparable expression levels (Odell et al., 1985; Lu et al., 2010; Tsugama et al., 2013; Amack and Antunes, 2020; Liang et al., 2023).

**Figure 7:**
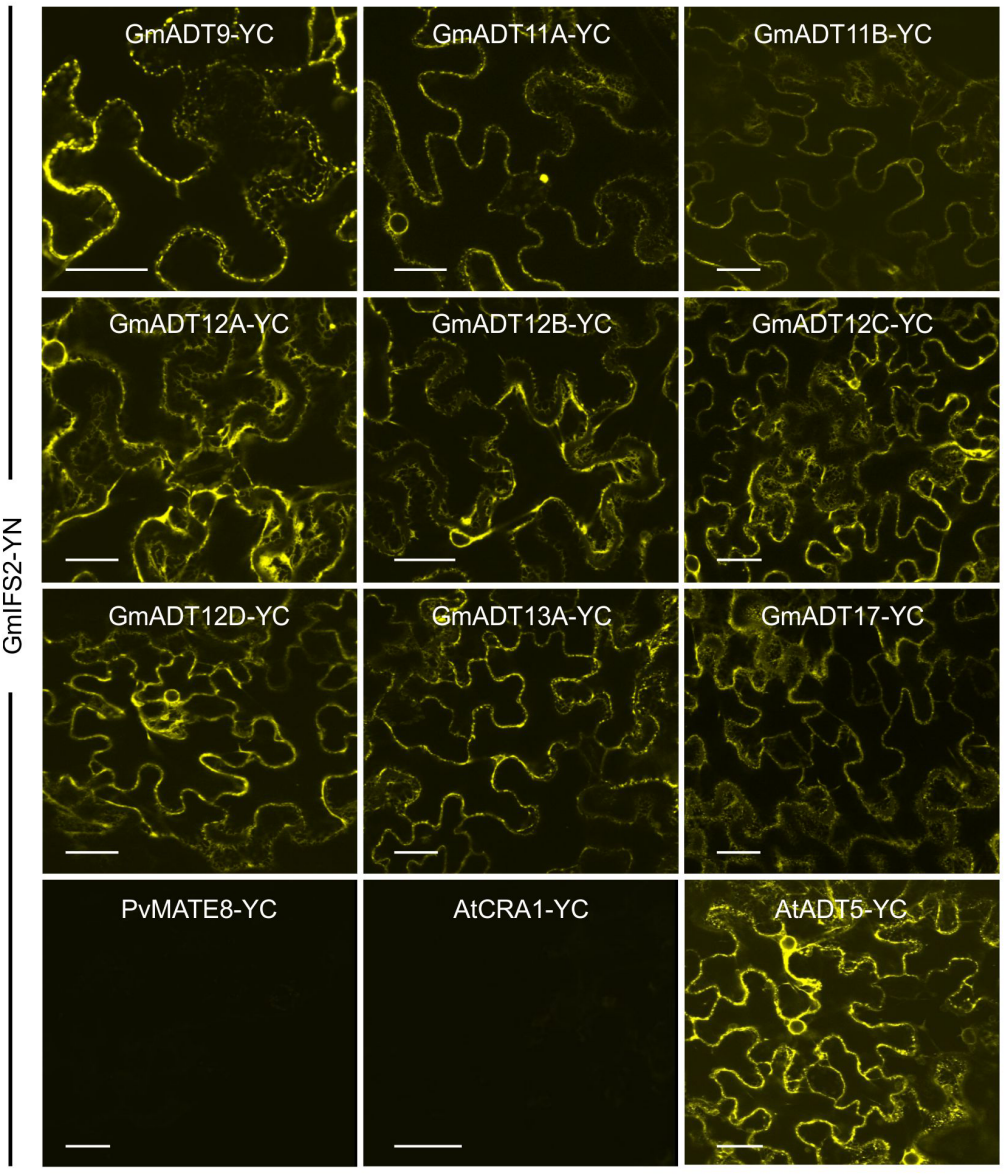
GmADTs and GmIFS2 interact *in planta* at the ER. Bi-directional interaction between GmADT isoforms and GmIFS2 by co-expression of translational fusions with N (YN)- or C (YC)- terminal fragments of YFP in *N. benthamiana* as assayed by BiFC. Proximity of the proteins results in a YFP fluorescence and localization of interaction as monitored by confocal microscopy. The interaction between chloroplast-localized GmADT and ER-localized GmIFS2 appear to be localized to the ER. As representative negative controls, GmMATE8-YC or AtCRA1-YC co-infiltrated with GmIFS2 (YN) is shown. Scale bars represent 30 µM.

Each GmADT was translationally fused to the C-terminal half of YFP (GmADT-YC) and GmIFS2 to the N-terminal half of YFP (GmIFS-YN). GmADT-YC and GmIFS2-YN constructs were co-expressed in *N. benthamiana* leaves and the protein-protein interaction was monitored using confocal microscopy (**Figure 7**). Co-expression of GmADT and GmIFS2 constructs were also performed in reciprocal combination (GmADT-YN and GmIFS2-YC (**Supplementary Figure 1**). As shown in **Figure 7**, the detection of YFP fluorescence indicating either close proximity or direct interaction between each of the GmADT family members and GmIFS2 was confirmed. The observed reticulate pattern of fluorescence indicated that the interaction was occurring at the ER surface where GmIFS2 is localized. We also detected an interaction between GmIFS2 and an *Arabidopsis* ADT, AtADT5. Similar results were obtained for the reciprocal combinations (**Supplementary Figure 1**). As negative controls, we also tested GmIFS2 with proteins unrelated to isoflavonoid synthesis and specialized metabolism: a protoanthocyanidin transporter protein from common bean (**Figure 7**, PvMATE8) and an *Arabidopsis* seed storage protein CRUCIFERIN1 (**Figure 7**, AtCRA1). No signal was detected when co-expressing GmIFS2 with PvMATE8 or AtCRA1 (**Figure 7**).

**Figure 8:**
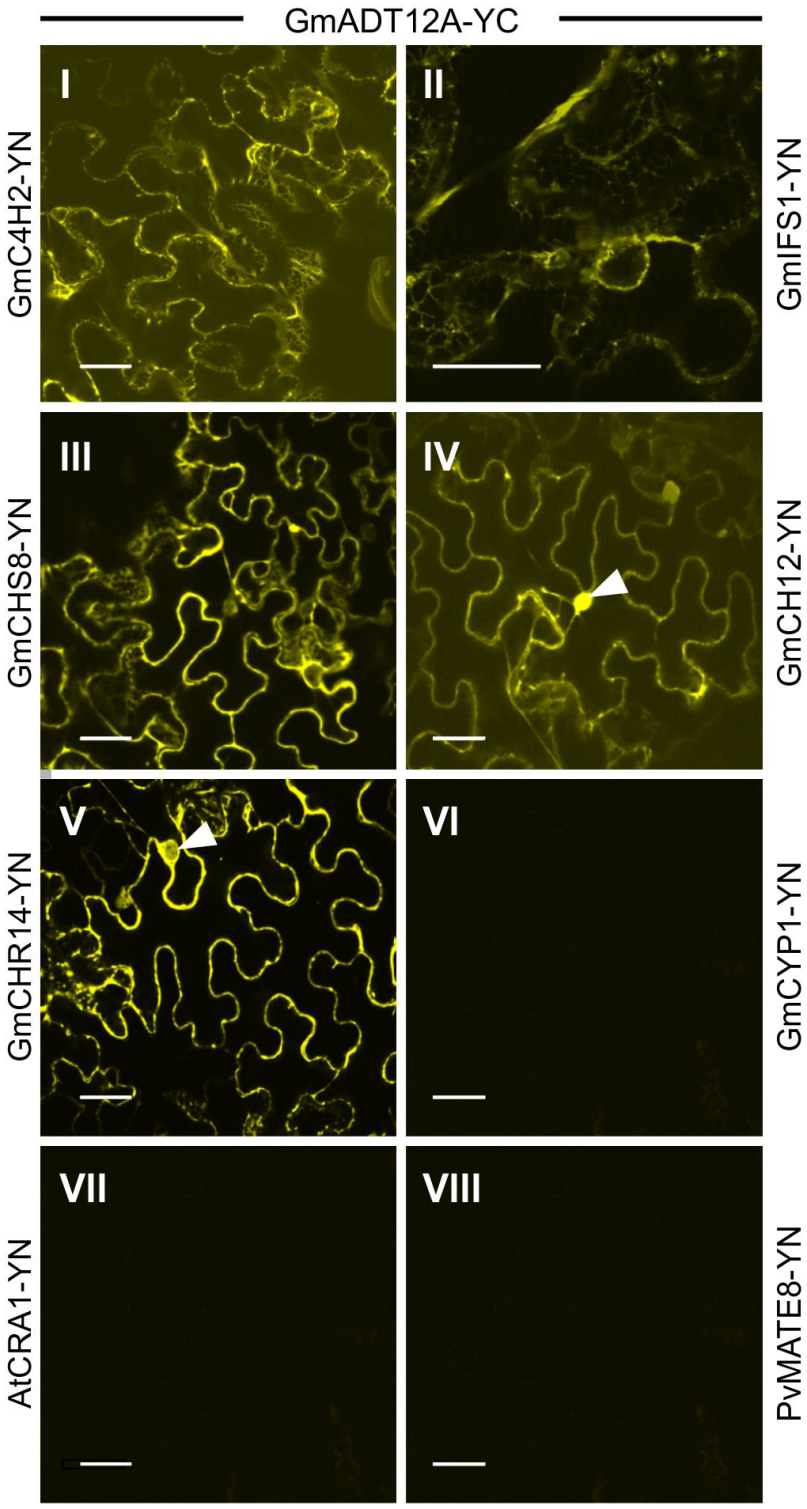
Interaction of GmADT12A with soluble and ER-localized isoflavonoid biosynthetic pathway enzymes by co-expression of translational fusions with N (YN)- or C (YC)- terminal fragments of YFP in *N. benthamiana* as assayed by BiFC. Proximity of the proteins results in a YFP fluorescence and localization of interaction as monitored by confocal microscopy. (I) and (II) The interaction between chloroplast localized GmADT12A and ER localized GmC4H2 and GmIFS1 appear to be localized to the ER. (III), (IV) and (V) GmADT12A and soluble enzymes GmCHS8, GmCHR14 and GmCHI2 appear to be localized to the cytoplasm and nucleus. White arrow heads point to nuclei. (VI), (VII) and (VIII) GmCYP1 is a nucleo-cytosolic protein (Mainali et al., 2017). No interaction was observed between GmADT12A and GmCYP1 or AtAtCRA1 or PvMATE8 (negative control). Scale bars represent 30 µM.

To investigate if GmADTs interact with the soluble enzymes of the (iso)flavonoid pathways, interaction between GmADT12A and GmCHS8, GmCHR14, GmCHI2 were also assayed by BiFC (**Figure 8**). GmADT12A was chosen as a representative ADT based on its putative involvement in the isoflavonoid metabolon (Dastmalchi et al., 2016). We also evaluated the interaction between GmADT12A and two other isoflavonoid ER anchors, GmC4H2 and GmIFS1 (Dastmalchi et al., 2016; Khatri et al., 2023). The interaction of GmADT12A with GmC4H2 and GmIFS1 was restricted to the surface of the ER (**Figures 8I,II**). Interestingly, the interaction of GmADT12A with soluble nucleo-cytoplasmic-localized proteins GmCHS8, GmCHR14 and GmCHI2 was detected in both the nucleus and cytosol (**Figures 8III-V**). No interaction was observed between GmADT12A and GmCYP1, AtCRA1 or PvMATE8 (**Figures 8VI–VIII**). No discrepancies were observed when testing the reciprocal combinations (**Supplementary Figure 2**).

### GmADTs with PDT activity

Sequence analysis suggested that some members of the GmADT family may have PDT activity, specifically GmADT9, GmADT11B, GmADT12C, GmADT12D and GmADT17 (**Figure 3**). To assess PDT activity of the GmADTs, the *pha2* yeast complementation assay (Bross et al., 2011) was performed. The *S. cerevisiae pha2* strain lacks the endogenous PDT protein, and can be complemented by a GmADT with PDT activity. Further, the *pha2* assay is inducible by galactose. Thus, only in the prescence of galactose GmADTs are expressed, and any GmADT with PDT activity will synthesize Phe and cells will grow without Phe supplementation.

**Figure 9:**
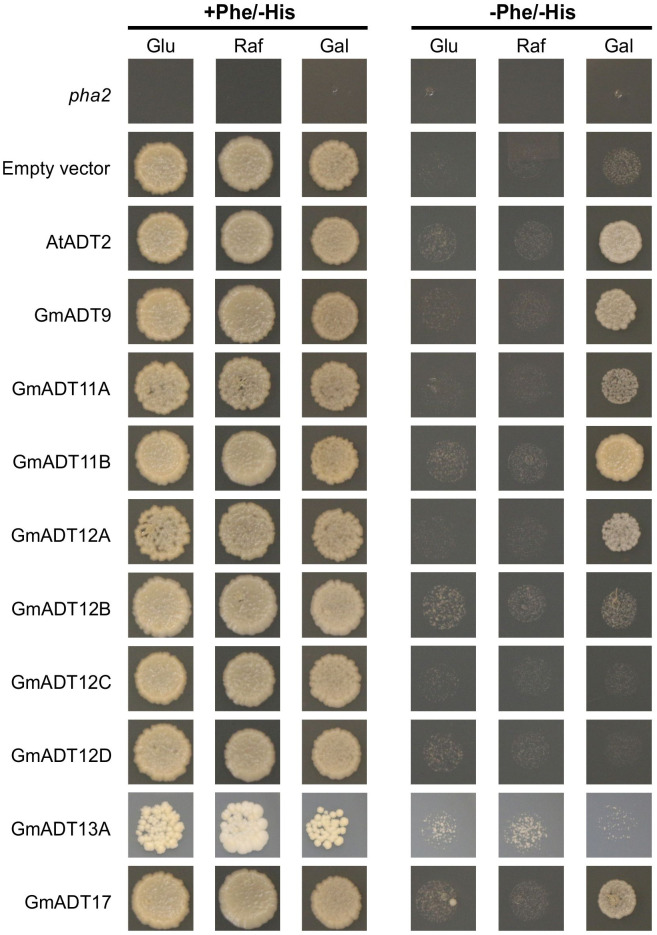
*pha2* complementation analysis of GmADTs. GmADTs were expressed in the yeast *pha2* strain and spotted onto SD media supplemented with either glucose (Glu), raffinose (Raf) or galactose (Gal). Media with each carbon source were also prepared either with or without phenylalanine (Phe). *S. cerevisiae pha2* strains transformed with GmADT were diluted to a final density of 5 x 10^4^ cells/mL and then equal volume of each was plated. Shown are representative growth spots, with images taken after 13 days of incubation. As negative controls, untransformed *pha2* strain and the *pha2* strain transformed with the empty expression vector were included. AtADT2 was used as a positive control. GmADT9, GmADT11A, GmADT11B, GmADT12A and GmADT17 complemented the *pha2* strain, demonstrating these GmADT enzymes possess PDT activity.

The untransformed *pha2* strain and *pha2* transformed with an empty vector were used as negative controls, and they do not complement the strain (**Figure 9**, *pha2* and empty vector). As a positive control, AtADT2 was used as it was previously shown to have PDT activity (Cho et al., 2007; Bross et al., 2011). As expected, no growth was observed when cells were grown with glucose or raffinose as carbon sources. When induced with galactose, GmADT9, GmADT11A, GmADT11B, GmADT12A and GmADT17 complemented the *pha2* strain, indicating these GmADT isoforms have PDT activity (**Figure 9**).

Next, Western blots were performed to confirm that all 6×His-tagged GmADTs were expressed in the *pha2* yeast strain (**Supplementary Figure 3**). Total soluble protein was extracted (Kushnirov, 2000), and equal volumes of extract were analyzed by SDS-PAGE. Coomassie staining was performed to ensure protein was successfully extracted (**Supplementary Figure 3**), and purified 6×His-GmCHR14 was used as a positive control. Signals from GmADT9, GmADT11A, GmADT11B, GmADT12A and GmADT13A were observed, however the remaining GmADTs (GmADT12B, GmADT12C, GmADT12D, and GmADT17) were not detected (**Supplementary Figure 3**). While GmADT17 appeared to be expressed sufficiently to complement the *pha2* strain, we cannot definitively conclude that GmADT12B, GmADT12C, and GmADT12D lack PDT activity, as they do not appear to be expressed in yeast.

## Discussion

Plant specialized metabolism is a tightly regulated network of overlapping pathways with many participating enzymes, all being controlled temporally during development, in response to external stimuli, and spatially in different plant tissues and subcellular organelles. Many of the enzymes involved in these biosynthetic pathways are encoded by multi-gene families (Boudet, 2007), and can direct carbon flux toward specific metabolic pathways (Cannon et al., 2004; Corea et al., 2012b). In the present study, we identified nine members of the *GmADT* family, examined their tissue-specific expression, and subcellular localization. We determined that five of the nine GmADTs possess PDT activity in the *in vivo pha2* complementation assay. We further showed that GmADTs interact with GmIFS2 *in vivo.* In particular, GmADT12A exhibits PDT activity, is expressed in root tissue and interacts with GmIFS2, substantiating its involvement in the isoflavonoid metabolon (Dastmalchi et al., 2016).

### Paleopolyploidy and GmADTs

There have been multiple whole genome duplications (WGDs) in the evolutionary history of soybean. Aside from the ancient WGD shared by all angiosperms (Young and Bharti, 2012), soybean has undergone two more recent WGDs: one just prior to radiation of legumes (~58-59 million years ago), and one *Glycine*-specific WGD that ocurred ~13-14 million years ago (Shoemaker et al., 2006; Schmutz et al., 2010; Yuan and Song, 2023). As a result, soybean has many duplicated genes, including the *GmADT* family members. There is often a period of gene loss following WGDs leading to the pseudogenization and loss of the redundant genes, but neofunctionalization of duplicates can also occur (Lynch and Force, 2000; Young and Bharti, 2012). Our data demonstrate that most of the *GmADT*s we identified encode functional proteins, indicating the GmADT isoforms are not fully redundant. The *GmADT*s are expressed in all soybean tissues, however they have unique temporal and spatial expression patterns, suggesting they are differentially regulated, and could differentially contribute to phenylpropanoid and (iso)flavonoid biosynthesis. *GmADT9* and *GmADT17* have diverged the most in sequence, sharing only ~50-63% identity with the other GmADTs at the nucleotide level and protein levels. Generally, GmADT isoforms that share high sequence identity, such as *GmADT11A* and *GmADT12B*, had similar expression profiles reflecting their shared evolutionary history. Though *GmADT11B* and *GmADT12D* share 93% nucleotide sequence identity, their expression patterns differ in the nodules, stem and throughout seed development, suggesting differences in regulation of gene expression and perhaps different contributions to specialized metabolism between these closely related isoforms. ADT isoforms from *Arabidopsis* have also been shown to be differentially expressed and contribute uniquely to metabolism (Corea et al., 2012b; Chen et al., 2016; Muhammad et al., 2023). For example, *GmADT11A*, *GmADT12A*, *GmADT12B*, and *GmADT13A* exhibited high mRNA expression in stems (**Figure 5**), and these GmADTs group with AtADT4 and AtADT5 in subgroup I (**Figure 4**). Of the six isoforms of the AtADT family, AtADT4 and AtADT5 contributed greatly to lignin production in stem tissue (Corea et al., 2012a) suggesting a role in lignin production for *GmADT11A*, *GmADT12A*, *GmADT12B*, and *GmADT13A*. El-Azaz and colleagues (2018) demonstrated that *AtADT2* expression was crucial for normal seed development in *Arabidopsis*. Further, the *adt2* seed phenotype could be rescued by both ADT and PDT activity, by expressing either *AtADT3* or *PHA2*, the PDT from *S. cerevisiae* (El-Azaz et al., 2018). *GmADT11B* and *GmADT12D* are the highest expressed soybean isoforms in seeds that also group with AtADT2 in subgroup II, indicating they may also have an important role in seed development. GmADT12D and GmADT17 are the most highly expressed in the nodules (**Figure 5**), suggesting these isoforms play an important role in the nodulation process. Taken together, the soybean *GmADT* have unique expression patterns that allows the isoforms to differentially contribute to Phe biosynthesis and ultimately specialized metabolism.

A consquence of WGDs is the alteration entire genetic networks, which can provide short-term advantages to biotic and abiotic stress tolerance (Van de Peer et al., 2017). In plants, many genes involved in stress responses and development are also present as large gene families (Cannon et al., 2004; Pan et al., 2022). In addition, the legume-specific WGD is suggested to have had a profound effect on nodulation and symbiosis (Young et al., 2011). Further, duplications in the flowering plant lineage has lead to a clade of allosterically deregulated ADT enzymes (referred to as type-II), and at least one member of this clade is present in most angiosperm genomes (El-Azaz et al., 2022). Type-I ADTs are tightly allosterically regulated by Phe, and include PpADT-G, AtADT1, GmADT9, GmADT11B and GmADT17. Type-II ADTs are much more loosely regulated, and include PpADT-C, AtADT4, and the remaining GmADT isoforms. These type-II isoforms are though to contribute to the accumulation of large quantities of Phe in the cell, which can potentially be shuttled toward specialized metabolite synthesis (El-Azaz et al., 2022; Muhammad et al., 2023). As isoflavonoids are a diverse class of compounds important for establishing symbioses with *Rhizobia*, a large family of differentially regulated and loosely allosterically regulated *ADT*s may allow more flexible control over (iso)flavonoid synthesis, in addition to some degree of functional redundancy conferred by large gene families (Kafri et al., 2009; El-Azaz et al., 2022; Muhammad et al., 2023).

### GmADTs interact with the isoflavonoid metabolon

Most ADT enzymes characterized to date are localized to the chloroplast, specifically to stromules (Rippert et al., 2009; Maeda et al., 2010; El-Azaz et al., 2016; Bross et al., 2017). However, some ADT isozymes localize to the nucleus or cytosol, for example, AtADT5 and AtADT6, respectively (Bross et al., 2017). We determined that the GmADTs were all localized to stromules. Since all nine GmADTs have an N-terminal chloroplast transit peptide, this suggests all GmADTs participate in phenylalanine biosynthesis *via* the well described chloroplast localized arogenate pathway. This is not surprising, as most plants described to date have a family of plastidic ADTs to convert arogenate to Phe (Jung et al., 1986; Ehlting et al., 2005; Cho et al., 2007; Rippert et al., 2009; Maeda and Dudareva, 2012; El-Azaz et al., 2016), and the GmADTs are similar in sequence to other characterized plant ADTs.

However, we observed interactions with GmIFS2 for all nine GmADTs that occur at the ER. In addition, GmADT12A also interacts both GmC4H2 and GmIFS1 at the ER membrane, GmCHS8 in the cytosol, and with GmCHI2 and GmCHR14 in the cytosol and the nucleus (**Figure 8**). The interaction of GmADT12A with GmCHI2 and GmCHR14 in the nucleus adds to the growing body of evidence that has detected (iso)flavonoid synthesis enzymes in the nucleus. Nucleo-cytoplasmic localizations have previously been reported for both GmCHI2 and GmCHR14 (Dastmalchi and Dhaubhadel, 2015; Sepiol et al., 2017). In addition, nuclear localization of CHI and CHS enzymes have also been reported in *Arabidopsis* and grapevine (Saslowsky et al., 2005; Wang et al., 2016; Watkinson et al., 2018; Wang et al., 2019). Localization of the (iso)flavonoid biosynthetic machinery in nucleus would allow direct deposition of these compounds in the nucleus (Saslowsky et al., 2005), however the role of (iso)flavonoids in the nucleus is not entirely clear. As flavonoids can bind DNA and have been shown to bind histones, regulation of gene expression by binding DNA associated proteins or DNA directly have been suggested as possible roles for these compounds in the nucleus (Hodek et al., 2002; Ramadass et al., 2003). Alternatively, the GmADTs and other (iso)flavonoid synthesis enzymes in the nucleus could have a secondary role there in regulating gene expression as retrograde signals (Isemer et al., 2012).

It is unlikely that the detected interaction is due to overpression of both GmIFS2 and the GmADTs, as no interaction was detected with PvMATE8 or AtCRA1 using the same detection system, indicating the interaction is specific to the GmADTs and GmIFS2. Furthermore, the same promoter was used for both detection of subcellular localization and protein-protein interactions, indicating the difference in GmADT localization is not soley due to overexpression using the 35S promoter. In addition, it has been shown that subcellular localization patterns detected in tobacco with a 35S promoter are indicative of what happens in the native organism. For example, AtADT5 nuclear localization was first determined in tobacco and was then confirmed when expressed under its native promoter in *Arabidopsis* (Bross et al., 2017), indicating the alternate nuclear localization was not an articact of expression in tobacco.

The current data substantiate our previous investigation of the isoflavonoid metabolon that was performed using the soybean hairy roots expressing GmIFS2-YFP, and GmADT12A and GmADT13A were pulled down in Co-IP analysis (Dastmalchi et al., 2016). Among the members of GmADT family, root-specific expression was only observed for *GmADT12A* and *GmADT13A* (**Figure 5**) which is consistent with only these two isoforms being identified by the Co-IP analysis. As the GmADTs are differentially expressed, isoflavonoid metabolon composition may vary depending on the GmADT isoform expressed in a given tissue. It has also been suggested that there could be more than one metabolon dedicated to the synthesis of different metabolites, given the availability of enzyme isoforms and their substrate preferences (Ehlting et al., 1999; Winkel, 2004; Li et al., 2015). One also needs to consider that in addition to tissue-specific expression of isoforms, metabolons are inherently transient and dynamic in nature. Consequently, there could be a high turnover of GmADT isoforms participating in the isoflavonoid metabolon, providing flexibility in metabolon assembly, substrate sequestering, metabolic channeling potential, and stress response (Winkel, 2002; Moller, 2010; Hawes et al., 2015).

### PDT activity of GmADTs

Most plant ADT families have least one isoform with predicted or demonstrated PDT activity, suggesting that both the prephenate and arogenate pathways are active in plants. It is thought that the Phe is predominantly synthesized *via* the arogenate pathway in the plastids. However, cytosolic PhPPY-AT and PhCM isoforms have been characterized (Yoo et al., 2013; Qian et al., 2019), and a cytosolic prephenate pathway has been described in *Petunia hybrida* (Qian et al., 2019). The possible advantages associated with the prephenate pathway, or the degree to which the prephenate pathway contributes to Phe synthesis is still unclear.

We determined that five GmADT isoforms had detectable PDT activity using the *pha2* complementation test: GmADT9, GmADT11A, GmADT11B, GmADT12A and GmADT17. As GmADT9 and GmADT17 fall within subgroup I, and GmADT11B in subgroup II in the ADT/PDT phylogenetic tree (**Figure 4B**), this result is not surprising. These three GmADT isoforms contain the critical Ala314 residue in the PAC domain that confers PDT activity to PpADT-B and PpADT-G (El-Azaz et al., 2016). Interestingly, GmADT11A and GmADT12A were also found to have PDT activity, but neither isoform contains the critical Ala314 residue. In fact, GmADT11A and GmADT12A both cluster within subgroup III, with isoforms from *Arabidopsis* and *P. pinaster* that do not have PDT activity. While some key resiudes for PDT activity have been identified in the PAC domain, it was noted that mutations in this domain had a variety of outcomes on PDT and ADT activity (El-Azaz et al., 2016; El-Azaz et al., 2022). It is possible that other unidentified amino acid residues within the PAC domain are important in defining PDT activity in soybean and other plant species. Further, by sequence alone, GmADT12C and GmADT12D were expected to act as PDTs. However, these two GmADT isoforms were among the GmADT proteins not well expressed in the *pha2* strain. As such, they could still have PDT activity if assessed using another method. We expect all the GmADTs to act as ADTs, given the sequence similarity to other plant ADTs, though we did not characterize ADT activity here. Determining the kinetics of the GmADTs with both arogenate and prephenate as a substrate should also be investigated in future, as it has been demonstrated that the *Arabidopsis* and *Petunia* ADT/PDTs prefer arogenate over prephenate as a substrate (Cho et al., 2007; Maeda et al., 2010). The proportion of phenylalanine synthesized via either pathway could also be tissue-specific, depending on whether the *ADT* isoform expressed in a given tissue has PDT activity.

### Trans-organelle continuity

**Figure 10:**
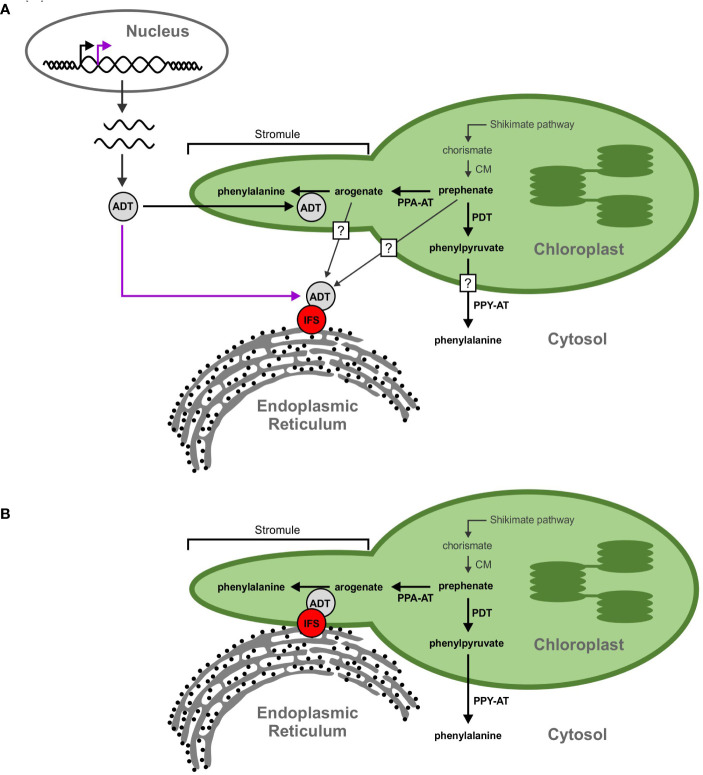
Proposed models for the GmADT-GmIFS2 interaction. **(A)** Full length ADT proteins contain an N-terminal transit peptide that directs them to the chloroplast (black arrow). However, alternate transcriptional start sites could result in a protein that lacks the transit peptide and remains in the cytosol (purple arrow). The cytosolic ADT protein would be available to interact with IFS and participate in the isoflavonoid metabolon. Substrates (prephenate, arogenate, phenylpyruvate, etc) could be transported out of the chloroplast, or they could passively diffuse across the chloroplast membrane, facilitated by the larger surface area of stromules. Prephenate could also be synthesized directly in the cytosol by cytosolic isoforms of CM. **(B)** Transorganelle continuity is another model allowing to explain the interaction between ADT and IFS proteins. The ER is a dynamic organelle that has been shown to interact with other organelles, namely the mitochondria and nucleus. Interactions between the ER and chloroplast membranes could bring the isoflavonoid metabolon and ADTs in close proximity.

As the cytoplasmic isoflavonoid metabolon assembles on the cytosolic face of the ER, we initially hypothesized that participating GmADTs are cytosolic, but no cytosolic GmADT isoforms were observed in this study when expressed alone. This begs the question, how can plastid-localized enzymes interact with a cytosolic metabolon? One possibility is that some of the *GmADT*s contain alternative transcriptional start sites, as seen for *PhADT3* (Qian et al., 2019), resulting in ADTs lacking a transit peptide that localize in the cytosoplasm (**Figure 10A**). Having transcript variants, with or without transit peptides, could allow differential contribution in response to various stress conditions or signaling cascades like nodulation induction. Alternatively, the full-length GmADTs could also associate with the metabolon in the cytosol prior to their transit to the chloroplast.

Another explanation for the involvement of GmADTs in the isoflavonoid metabolon is trans-organelle continuity (**Figure 10B**). The ER is a dynamic organelle (Allen and Brown, 1988; Bayer et al., 2017; Kriechbaumer and Brandizzi, 2020), and there is evidence that the ER can associate with various organelles in the cell, including the chloroplast (Schattat et al., 2012; Phillips and Voeltz, 2016; Barton et al., 2018). It has been demonstrated in mammalian and plant cells that the ER forms contacts with peroxisomes, mitochondria, the plasma membrane and plasmodesmata (Hawes et al., 2015; Bayer et al., 2017; Perico and Sparkes, 2018; Li et al., 2022). The ER has also been shown to be involved in nuclear envelope formation and reshaping during cell division, and may facilitate protein localization to the inner nuclear membrane (Anderson and Hetzer, 2007; Blackstone, 2017; Ungricht and Kutay, 2017).

Multiple contact sites have been demonstrated between the ER membrane and chloroplasts (Griffing, 2011; Block and Jouhet, 2015), and it has even been suggested that the ER and chloroplast membranes can become contiguous (Whatley et al., 1991). ER morphology changes have also been shown to correlate with the growth and retraction of stromules (Schattat et al., 2011). Stromules have been reported to expedite the transport and/or diffusion of small molecules and proteinsbetween the plastid and surrounding cellular compartments, and have been shown to be involved in various signaling and defense responses (Gray et al., 2001; Hanson and Hines, 2018; Perico and Sparkes, 2018), as well as plastid-to-plastid contact sites, and possible contacts with other organelles (Kwok and Hanson, 2004a; Erickson et al., 2017). It has also been demonstrated that ER lumen localized enzymes have access to tocopherol substrates at the inner plastid membrane (Mehrshahi et al., 2013), making trans-orgnalle continuity a compelling argument for GmADT access to the (iso)flavonoid metabolon. Stromule localization of GmADT proteins places them in a dynamic sub-plastidial environment, facilitating interactions with the ER, and subsequent association with the (iso)flavonoid metabolon. GmADT involvement in the (iso)flavonoid metablon would directly link flux toward Phe biosynthesis to the synthesis of specialized metabolites.

Organelle interactions offer a variety of advantages. Substrates do not need to be exported from organelles and diffused across the cytosol. Required substrates and intermediates are sequestered, limiting byproduct formation and increasing pathway efficiency (Moller, 2010; Zhang and Fernie, 2021). Regulating organelle contacts could also provide another avenue to regulate flux through pathways, in addition to transcriptional and translational regulation of enzymatic components.

### Conclusions and outlook

We have identified nine *GmADT* family members and demonstrated their interaction with GmIFS2 and other (iso)flavonoid metabolon enzymes using an *N. benthamiana* BiFC expression system. While we previously reported the interaction of GmADT12A and GmADT13A with GmIFS2 in soybean roots, confirmation of the interaction between GmIFS2 and the other GmADTs in soybean tissue is still required. In addition, we have demonstrated that at least five of the nine GmADTs have PDT activity by yeast complementation analysis in the *pha2* strain. However, there are still questions surrounding the *GmADT* family members and their involvement in (iso)flavonoid synthesis. In this work, we did not assess the ADT activity of the soybean ADTs. While they appear to be homologous to other plant ADTs, and share many of the sequences defining ADT activity and regulation, quantifying the ADT activity of the GmADT family is still necessary. Further, the factors influencing flux between the arogenate pathway and the prephenate pathway, and how GmADTs contribute phenylalanine to the (iso)flavonoid metabolon are yet unknown. Finally, determining the extent to which tissue-specific expression influences the (iso)flavonoid metabolon and ADT isoform involvement would provide insights into the differential regulation and diverging functions of gene family members.

## Data availability statement

The original contributions presented in the study are included in the article/**Supplementary Material**. Further inquiries can be directed to the corresponding author.

## Author contributions

EC: Data curation, Investigation, Formal analysis, Methodology, Writing – original draft. NI: Data curation, Methodology, Software, Validation, Writing – review & editing. KP: Writing – review & editing, Investigation. KK: Investigation, Writing – review & editing, Validation. RS: Investigation, Writing – review & editing. SK: Writing – review & editing, Supervision. SD: Supervision, Writing – review & editing, Conceptualization, Data curation, Funding acquisition, Investigation, Project administration, Resources, Validation, Visualization.

## Supplementary material

Supplementary Figure 1 GmADT and GmIFS2 interact *in planta* at the ER. Reciprocal combination of GmADT isoforms and GmIFS2 (as shown in )and their interaction by co-expression of translational fusions with N (YN)- or C (YC)- terminal fragments of YFP in *N. benthamiana* as assayed by BiFC.

Supplementary Figure 2 GmADT12A-YN and isoflavonoid biosynthetic enzymes-YC interaction (reciprocal combination of ).

Supplementary Figure 3 Western blot detection of *Gm*ADTs in *S. cerevisiae pha2.* **(A)** GmADTs- his were expressed in the yeast *pha2* strain and detected by western blotting analysis using anti-his antibody. **(B)** Coomassie stained gel of total protein under non-inducing (glucose) condition. **(C)** Coomassie stained gel of total protein under inducing (galactose) condition.
