# Supplementary figures and images for: Soybean AROGENATE DEHYDRATASES (GmADTs): involvement in the cytosolic isoflavonoid metabolon or trans-organelle continuity?
Source: Front Plant Sci. 2024 Jan 23;15:1307489. doi: 10.3389/fpls.2024.1307489 (PMC10845154; doi:10.3389/fpls.2024.1307489)

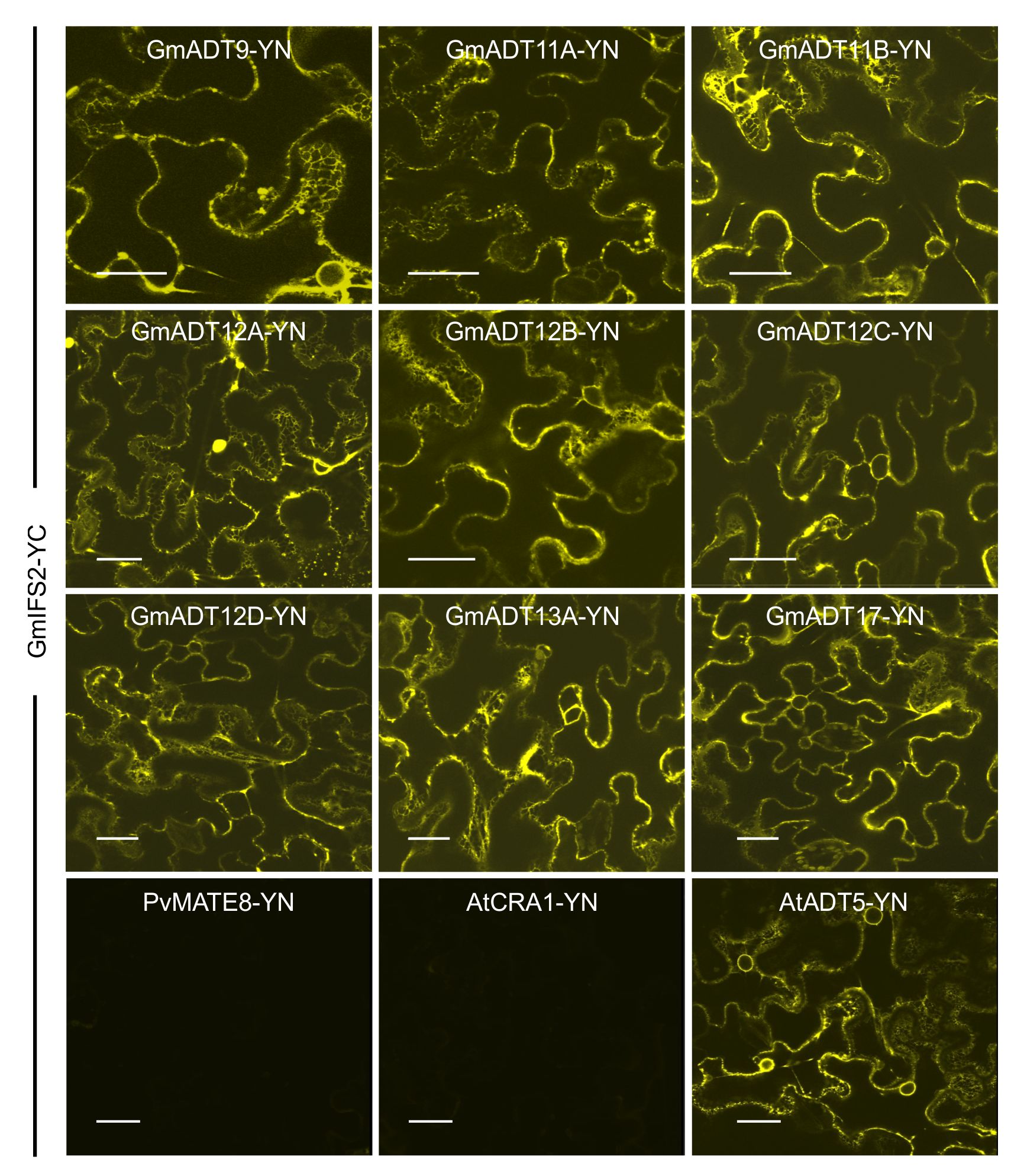

Supplement: Supplementary Figure 1 — GmADT and GmIFS2 interact in planta at the ER. Reciprocal combination of GmADT isoforms and GmIFS2 (as shown in )and their interaction by co-expression of translational fusions with N (YN)- or C (YC)- terminal fragments of YFP in N. benthamiana as assayed by BiFC. [file Image_1.jpeg]

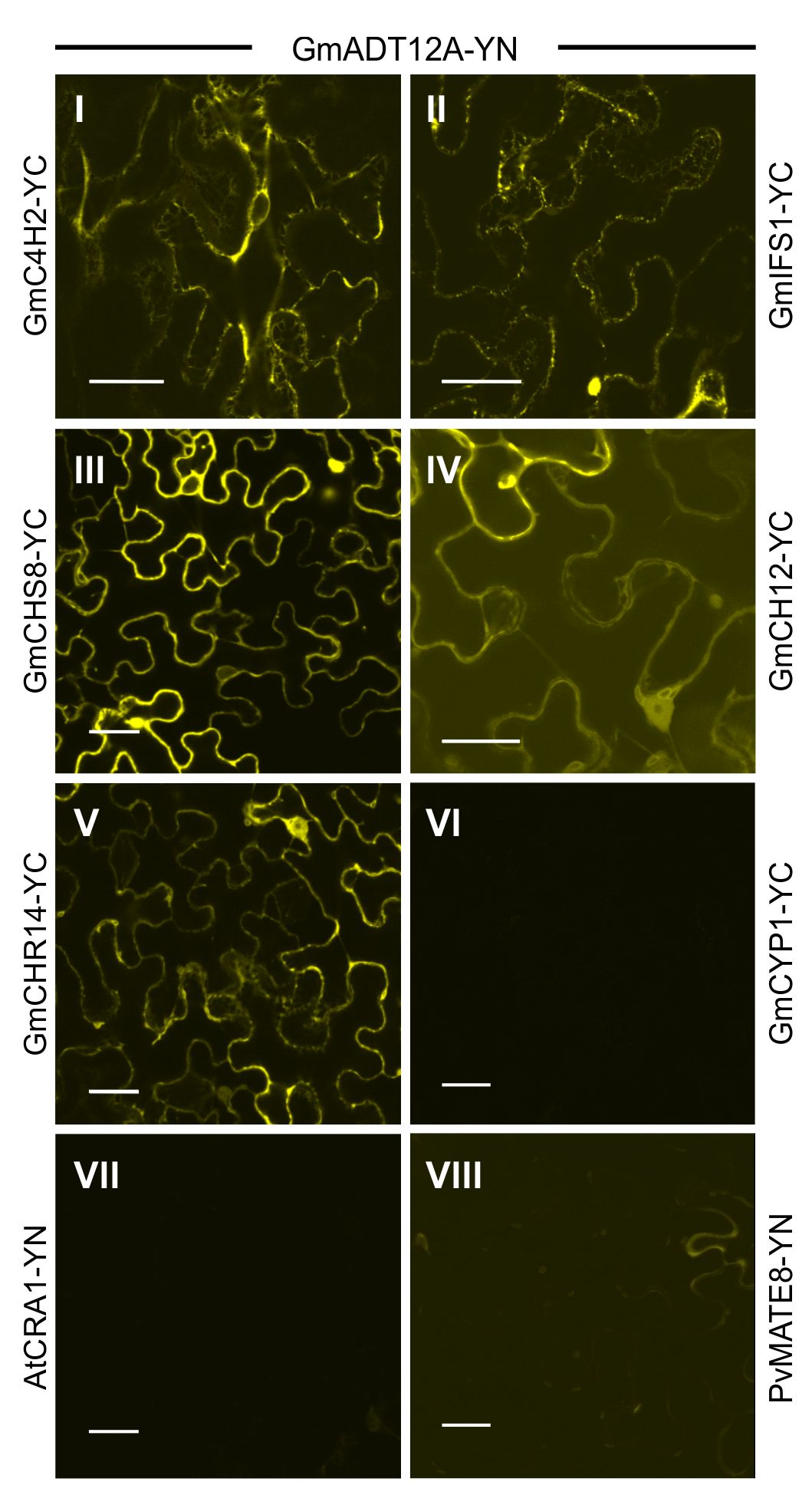

Supplement: Supplementary Figure 2 — GmADT12A-YN and isoflavonoid biosynthetic enzymes-YC interaction (reciprocal combination of ). [file Image_2.jpeg]

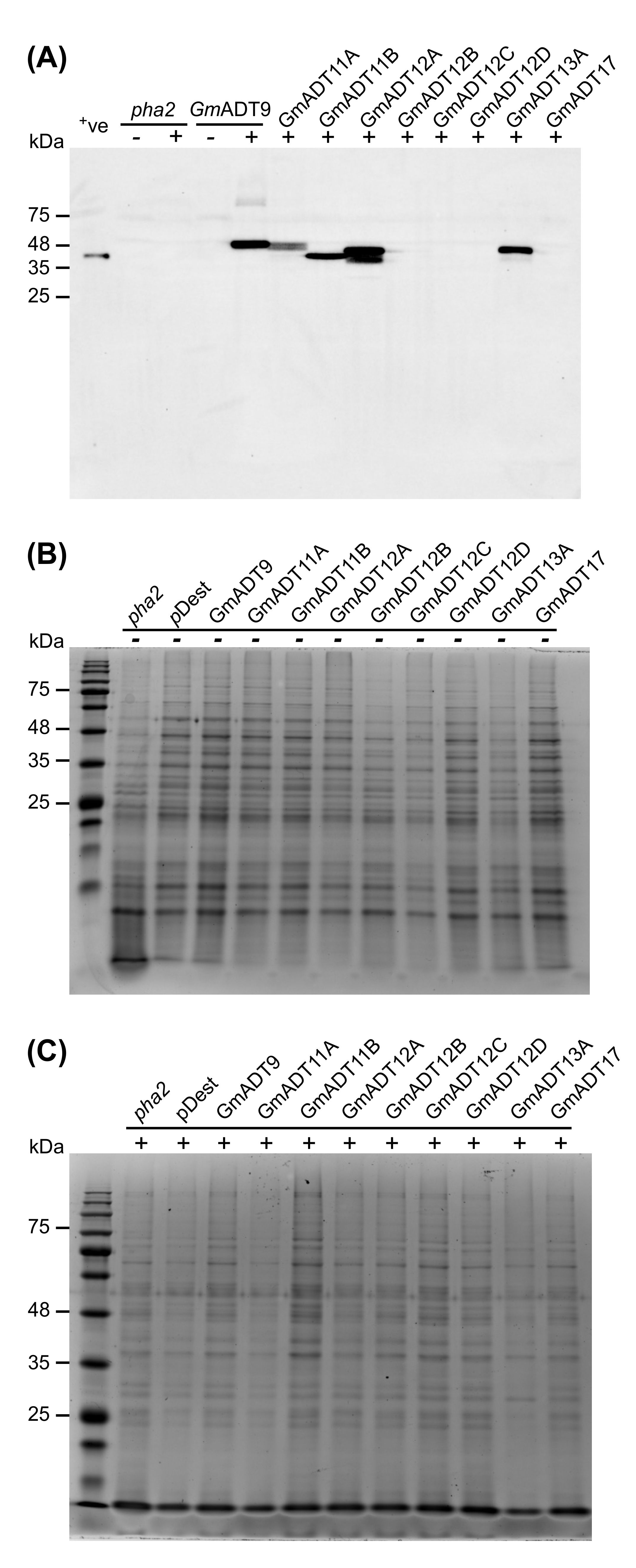

Supplement: Supplementary Figure 3 — Western blot detection of GmADTs in S. cerevisiae pha2. (A) GmADTs- his were expressed in the yeast pha2 strain and detected by western blotting analysis using anti-his antibody. (B) Coomassie stained gel of total protein under non-inducing (glucose) condition. (C) Coomassie stained gel of total protein under inducing (galactose) condition. [file Image_3.jpeg]
