# Supplementary material for: Soybean AROGENATE DEHYDRATASES (GmADTs): involvement in the cytosolic isoflavonoid metabolon or trans-organelle continuity?
Source: Front Plant Sci. 2024 Jan 23;15:1307489. doi: 10.3389/fpls.2024.1307489 (PMC10845154; doi:10.3389/fpls.2024.1307489)
Supplement: Supplementary file 4 [file Table_1.docx]

**_Table S1._** _List of primers used in gene cloning for subcellular localization, protein-protein interaction and PDT assay._

| **_Gene name_** | **_Primer Name_** | **_Primer Sequence (5’—3’)_** | **_Amplicon size (nt)_** | **_Use_** |
| --- | --- | --- | --- | --- |
| *_GmADT9_* | _GmGTADT9F_ | _GGGGACAAGTTTGTACAAAAAAGCAGGCTTCATGCGTGTGGTTGATCATCCT_ | _1212_ | _Gene cloning for subcellular localization and BiFC_ |
|  | _GmGTADT9R_ | _GGGGACCACTTTGTACAAGAAAGCTGGGTCCCATACCCGAAGAAATGTGGC_ |  |  |
| *_GmADT11A_* | _GmGTADT11AF_ | _GGGGACAAGTTTGTACAAAAAAGCAGGCTTCATGCAGACCCTCAATCAA_ | _1284_ |  |
|  | _GmGTADT11AR_ | _GGGGACCACTTTGTACAAGAAAGCTGGGTCATTTTGGCGCGGACAA_ |  |  |
| *_GmADT11B_* | _GmGTADTU4F_ | _GGGGACAAGTTTGTACAAAAAAGCAGGCTTCATGGCGGCATCGCGAATCGTG_ | _1155_ |  |
|  | _GmGTADTU4R_ | _GGGGACCACTTTGTACAAGAAAGCTGGGTCCGTCAAGCTAGTGTCCACAGGATA_ |  |  |
| *_GmADT12A_* | _GmGTADT12AF_ | _GGGGACAAGTTTGTACAAAAAAGCAGGCTTCATGCAGACTCTTTCGCC_ | _1275_ |  |
|  | _GmGTADT12AR_ | _GGGGACCACTTTGTACAAGAAAGCTGGGTCTTTAAATTTATCTCCCCGGGAGG_ |  |  |
| *_GmADT12B_* | _GmGTADT12BF_ | _GGGGACAAGTTTGTACAAAAAAGCAGGCTTCATGCAGACCCTCACCC_ | _1284_ |  |
|  | _GmGTADT12BR_ | _GGGGACCACTTTGTACAAGAAAGCTGGGTCATTTTGGCGCGGAGAAGA_ |  |  |
| *_GmADT12C_* | _GmGTADT12CF_ | _GGGGACAAGTTTGTACAAAAAAGCAGGCTTCATGGCTGTGACATCACCTCTTG_ | _1152_ |  |
|  | _GmGTADT12CR_ | _GGGGACCACTTTGTACAAGAAAGCTGGGTCTATGGTTGTATCTATGGGATAGCAG_ |  |  |
| *_GmADT12D_* | _GmGTADT12DF_ | _GGGGACAAGTTTGTACAAAAAAGCAGGCTTCATGGCTGCGTCGCGAATC_ | _930_ |  |
|  | _GmGTADT12DR_ | _GGGGACCACTTTGTACAAGAAAGCTGGGTCTACCTTTGTAAGGTTAATCTGACGC_ |  |  |
| *_GmADT13A_* | _GmGTADT13AF_ | _GGGGACAAGTTTGTACAAAAAAGCAGGCTTCATGCAGAGTCTTTCACCACC_ | _1272_ |  |
|  | _GmGTADT13AR_ | _GGGGACCACTTTGTACAAGAAAGCTGGGGTCTCTCCCCGGGAGGAA_ |  |  |
| *_GmADT13B_* | _GmGTADT13BF_ | _GGGGACAAGTTTGTACAAAAAAGCAGGCTTCATGCGTGTGGTTGATCATCCT_ | _642_ |  |
|  | _GmGTADT13BR_ | _GGGGACCACTTTGTACAAGAAAGCTGGGTCCCATACCCGAAGAAATGTGGC_ |  |  |
| *_GmADT17_* | _GmGTADT17AF_ | _GGGGACAAGTTTGTACAAAAAAGCAGGCTTCATGGCTCTTAAGGCTGTATC_ | _1197_ |  |
|  | _GmGTADT17AR_ | _GGGGACCACTTTGTACAAGAAAGCTGGGGTCTTAAGACACTGAACTTCTATAATACT_ |  |  |
| *_GmADT9_* | _GmADT9AGW-F_ | _GGGGACAAGTTTGTACAAAAAAGCAGGCTTCATGGCTCTCAAGGATTGTTCCATC_ | _1233_ | _Gene cloning for PDT assay_  _assay_ |
|  | _GmADT9AGWHis-R_ | _GGGGACCACTTTGTACAAGAAAGCTGGGTCTCAGTGGTGGTGGTGGTGGTGATCTGATGTATCTGTTTTATCCACCGG_ |  |  |
| *_GmADT11A_* | _GmGTADT11AF_ | _GGGGACAAGTTTGTACAAAAAAGCAGGCTTCATGCAGACCCTCAATCAA_ | _1305_ |  |
|  | _GmADT11AR-GWY2_ | _GGGGACCACTTTGTACAAGAAAGCTGGGTCCTAATGATGATGATGGTGATGATTTTGGCGCGGACA_ |  |  |
| *_GmADT11B_* | _GmGTADTU4F_ | _GGGGACAAGTTTGTACAAAAAAGCAGGCTTCATGGCGGCATCGCGAATCGTG_ | _1176_ |  |
|  | _GmADTU4R-GWY2_ | _GGGGACCACTTTGTACAAGAAAGCTGGGTCCTAATGATGATGATGGTGATGCGTCAAGCTAGTGTC_ |  |  |
| *_GmADT12A_* | _GmADT12AGW-F_ | _GGGGACAAGTTTGTACAAAAAAGCAGGCTTCATGCAGACTCTTTCGCCGCCT_ | _1296_ |  |
|  | _GmADT12AGWHis-R_ | _GGGGACCACTTTGTACAAGAAAGCTGGGTCCTAGTGGTGGTGGTGGTGGTGATCTCCCCGGGAGGAAGGTGT_ |  |  |
| *_GmADT12B_* | _GmGTADT12BF_ | _GGGGACAAGTTTGTACAAAAAAGCAGGCTTCATGCAGACCCTCACCC_ | _1305_ |  |
|  | _GmADT12BR-GWY2_ | _GGGGACCACTTTGTACAAGAAAGCTGGGTCCTAATGATGATGATGGTGATGATTTTGGCGCGGAGA_ |  |  |
| *_GmADT12C_* | _GmGTADT12CF_ | _GGGGACAAGTTTGTACAAAAAAGCAGGCTTCATGGCTGTGACATCACCTCTTG_ | _1173_ |  |
|  | _GmADT12CR-GWY2_ | _GGGGACCACTTTGTACAAGAAAGCTGGGTCCTAATGATGATGATGGTGATGTATGGTTGTATCTAT_ |  |  |
| *_GmADT12D_* | _GmGTADT12DF_ | _GGGGACAAGTTTGTACAAAAAAGCAGGCTTCATGGCTGCGTCGCGAATC_ | _951_ |  |
|  | _GmADT12DR-GWY2_ | _GGGGACCACTTTGTACAAGAAAGCTGGGTCCTAATGATGATGATGGTGATGAAGTACCTTTGTAAG_ |  |  |
| *_GmADT13A_* | _GmGTADT13AF_ | _GGGGACAAGTTTGTACAAAAAAGCAGGTTCATGCAGAGTCTTTCACCACC_ | _1293_ |  |
|  | _GmADT13AR-GWY2_ | _GGGGACCACTTTGTACAAGAAAGCTGGGTCCTAATGATGATGATGGTGATGGTCTCCCCGGGAGGA_ |  |  |
| *_GmADT17_* | _GmGTADT17AF_ | _GGGGACAAGTTTGTACAAAAAAGCAGGCTTCATGGCTCTTAAGGCTGTATC_ | _1218_ |  |
|  | _GmADT17AR-GWY2_ | _GGGGACCACTTTGTACAAGAAAGCTGGGTCCTAATGATGATGATGGTGATGGTTAAGACACTGAAC_ |  |  |
